# Exploring the Unseen: A Survey of Multi-Sensor Fusion and the Role of Explainable AI (XAI) in Autonomous Vehicles

**DOI:** 10.3390/s25030856

**Published:** 2025-01-31

**Authors:** De Jong Yeong, Krishna Panduru, Joseph Walsh

**Affiliations:** 1 IMaR Research Centre, Munster Technological University, Tralee, V92 CX88 Co. Kerry, Ireland; krishna.panduru@mtu.ie (K.P.); joseph.walsh@mtu.ie (J.W.); 2 School of Science, Technology, Engineering, and Mathematics, Munster Technological University, Tralee, V92 CX88 Co. Kerry, Ireland; 3 Lero—The Science Foundation Ireland Research Centre for Software, V92 NYD3 Limerick, Ireland

**Keywords:** autonomous vehicles, self-driving cars, multi-sensor fusion, explainability, explainable artificial intelligence (XAI), interpretability, perception, camera, lidar, radar

## Abstract

Autonomous vehicles (AVs) rely heavily on multi-sensor fusion to perceive their environment and make critical, real-time decisions by integrating data from various sensors such as radar, cameras, Lidar, and GPS. However, the complexity of these systems often leads to a lack of transparency, posing challenges in terms of safety, accountability, and public trust. This review investigates the intersection of multi-sensor fusion and explainable artificial intelligence (XAI), aiming to address the challenges of implementing accurate and interpretable AV systems. We systematically review cutting-edge multi-sensor fusion techniques, along with various explainability approaches, in the context of AV systems. While multi-sensor fusion technologies have achieved significant advancement in improving AV perception, the lack of transparency and explainability in autonomous decision-making remains a primary challenge. Our findings underscore the necessity of a balanced approach to integrating XAI and multi-sensor fusion in autonomous driving applications, acknowledging the trade-offs between real-time performance and explainability. The key challenges identified span a range of technical, social, ethical, and regulatory aspects. We conclude by underscoring the importance of developing techniques that ensure real-time explainability, specifically in high-stakes applications, to stakeholders without compromising safety and accuracy, as well as outlining future research directions aimed at bridging the gap between high-performance multi-sensor fusion and trustworthy explainability in autonomous driving systems.

## 1. Introduction

Autonomous vehicles (AVs), also known as self-driving vehicles, are at the forefront of technological innovation with the potential to transform and revolutionize transportation by improving road user safety, efficiency, and accessibility and reducing greenhouse gas emissions [1,2]. At the core of their operation lies the sophisticated capability to perceive, analyze, and respond to highly dynamic and complex driving environments in real time with minimal to no human intervention. An AV’s perception system relies on the integration of advanced proprioceptive and exteroceptive sensors, robust processing power, complex machine learning (ML) algorithms, and decision-making systems to analyze and interpret complex traffic situations, navigate through unpredictable conditions, and make real-time critical driving decisions autonomously [2]. In our previous research [3], we investigated the architecture of an autonomous driving system from both functional and technical perspectives, highlighting the key components and subsystems that facilitate AVs to operate efficiently based on system design and operational capabilities, specifically in the perception stage of self-driving solutions.

AVs are not limited to on-road applications such as highway driving and navigation or urban driving, nor to off-road environments in industries like agriculture, mining, and construction [4,5,6]. This technology extends to a broader range of domains, including maritime settings, where AVs are applied to manage self-navigating vessels, automated container handling, and logistic operations in container port terminals, et cetera, hence improving the safety and efficiency of port activities [7,8]. Whether operating in structured urban settings with well-defined road networks, navigating unstructured and rugged off-road terrains, or coordinating day-to-day logistical tasks within dynamic maritime settings, AVs face diverse operational challenges that demand advanced solutions. All these challenges require efficient and robust multi-sensor fusion and decision-making algorithms to ensure effective and reliable performance.

In AVs, sensors play a pivotal role in perceiving the surroundings and localization of the vehicle within its environment to perform dynamic driving tasks such as obstacle detection and avoidance, path planning, environmental awareness, response to unexpected road situations, et cetera [9,10]. This involves real-time collection and interpretation of large volumes of data (or measurements) from multiple proprioceptive and exteroceptive sensors, including vision cameras, radar, Lidar, ultrasonic sensor, Global Positioning System (GPS), Inertial Measurement Unit (IMU), et cetera. Table 1 below provides a summary of the commonly adopted proprioceptive and exteroceptive sensors in an AV. It outlines the specific types of sensor that are frequently used in autonomous driving systems to enable robust perception and localization across various operational contexts [11,12].

However, the composition of the sensor suite, which refers to the collection of sensors that are integrated into an AV, can vary significantly based on the intended use cases and their specific operational demands. In addition, the specific operational environment of AVs—whether it is on-road, off-road, or in specialized industrial settings—affects the type and arrangement of the sensors that are required to facilitate the perception, localization, and decision-making processes in an autonomous driving system. For example, on-road AVs such as self-driving cars [13] or trucks [14] that operate predominantly on highways and within urban environments often rely heavily on a combination of vision cameras, radar, and Lidars to ensure high-resolution and 360-degree environmental mapping; which are vital in environments where dense traffic and high-speed motion are involved. These sensors must be able to detect and track moving objects, interpret traffic signals, and respond to unpredictable behaviors from other road users.

**Figure 1:**
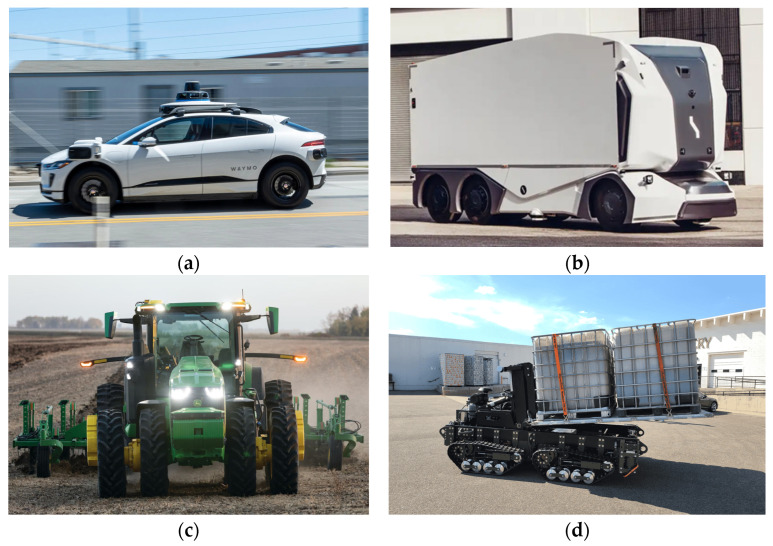
A visual representation of various examples of AVs specifically designed for both on-road and off-road applications. (**a**) Waymo self-driving taxis for ride-sharing services; (**b**) Einride autonomous truck for freight transportation and logistics; (**c**) John Deere autonomous tractor and tillage for agricultural activities and precision farming; (**d**) Stratom autonomous pallet loader for handling pallets. All images shown are provided by the following sources: [14,16,17,19].

In contrast, off-road AVs such as autonomous tractors and tillage (agriculture), autonomous pallet loaders (military and warehousing), automated rail mounted gantry (RMG) cranes (shipping yards), et cetera [15,16,17], may employ a different sensor configuration that incorporates robustness due to rugged environments, uneven surfaces, low-visibility conditions, or lack of clear infrastructures. In such cases, off-road AVs often incorporate specialized sensors like infrared cameras or thermal cameras to enhance visibility in dusty or low-light conditions [18]. Figure 1 below presents a visual depiction of various examples of AVs specifically designed for both on-road and off-road applications. The imagery exemplifies the diversity present within the category of AVs, highlighting how different designs and functionalities are tailored to meet the unique requirements of different operational environments.

**Figure 2:**
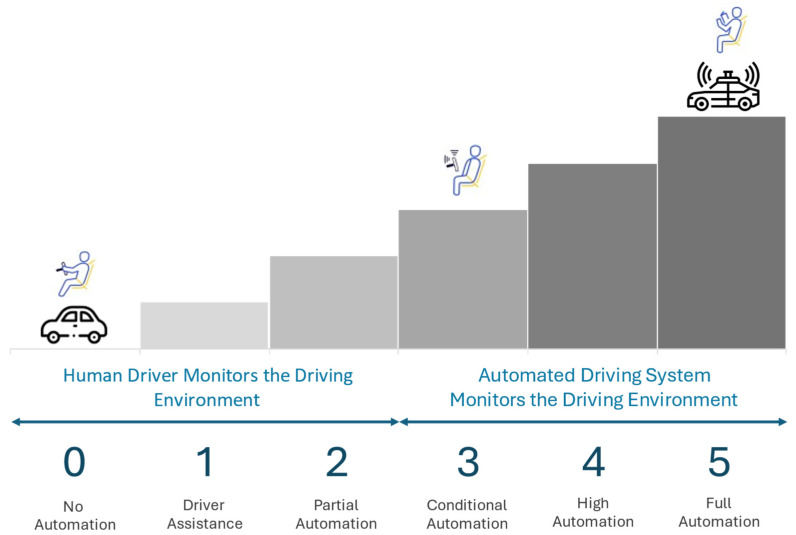
A visual summary of the SAE J3016:2021 standard, which categorizes the levels of driving automation in vehicles. Readers interested in the comprehensive description of the SAE J3016:2021 standard (latest revision) are advised to refer to the SAE International Blog Post [27]. The illustration shown was redrawn and modified based on the diagram in references [28,29].

The Society of Automative Engineers (SAE) introduced a standardized guideline to eliminate confusion in the terminology used to describe the varying levels of vehicle automation. It aims to promote clearer communication across industries, enhance risk assessment during system design, support the development of safety and regulatory frameworks, and build public trust and understanding of AV technologies [10,20]. Hence, its initiative has led to the publication of the SAE J3016 standard in 2014, which clearly classifies the levels of driving automation ranging from Level 0 (no automation) to Level 5 (full automation) [21], as illustrated in Figure 2. Current automation driving technologies have yet to reach their full potential and have remained at Level 2 (partial automation) for several years [10]. Nonetheless, it is important to highlight that Level 3 (conditional automation) automated driving systems are now being initiated into standard production by manufacturers such as Mercedes-Benz (S Class and EQS) and BMW (7 Series) in Germany, as well as in multiple regions across the United States (California and Nevada) [22,23,24,25]. Additionally, some manufacturers, such as Waymo’s commercial self-driving ride-sharing services [26], claim to have built vehicles with autonomy that are equivalent to Level 4 (high automation), as described in the SAE J3016 standard. In both on-road and off-road applications, the adoption of this standardized classification supports more coherent development pathways for multi-sensor fusion and explainable artificial intelligence (XAI), as it provides a transparent understanding of the driving system’s intended level of autonomy, decision-making responsibilities, and operational limitations.

A shared characteristic of an autonomous driving system, applicable to both on-road and off-road applications, is their reliance on multi-sensor fusion, a method that involves integration of data from multiple sensor types. This approach is essential for improving the overall perception and situational awareness of AVs, as it helps to address the limitations inherent in individual sensors operating in isolation and mitigate detection uncertainties. For instance, Lidar sensors are highly effective at providing precise, high-resolution depth information, though they are susceptible to adverse weather conditions. In contrast, radar sensors are more capable of detecting objects through fog or rain but may offer lower spatial resolution [11]. By integrating data from diverse sensor modalities such as exteroceptive sensors and proprioceptive sensors, multi-sensor fusion significantly enhances the accuracy, reliability, and robustness of the vehicle’s perception capabilities. Thus, such an approach enables AVs to achieve a more comprehensive understanding of the surroundings, facilitating more effective navigation in complex and dynamic environments [30,31].

Nonetheless, as the complexity of autonomous driving systems increases, especially with the integration of multiple sensor modalities, the decision-making processes guided by complex deep learning (DL) and ML algorithms often lead to a significant lack of transparency. While these DL and ML models are highly effective at generalizing across a wide range of driving scenarios and are renowned for their powerful ability to model complex patterns through sophisticated data representation, their inner workings and underlying decision-making logic often results in an inexplainable system [32]. Such systems are concerning in safety-critical applications, such as AVs, where the consequences of erroneous or suboptimal decisions can be severe. For example, in scenarios involving novel conditions or sophisticated driving environments, the inability to understand how or why an autonomous system has made a particular decision can lead to significant risks, including system failures, accidents, or even the loss of human life [33,34]. Hence, it is important to integrate explainability into the design of complex autonomous systems to enhance transparency, traceability, accountability, and trust among stakeholders [35].

This paper builds upon and extends the research presented in our previous publication [11], broadening the scope to deliver an in-depth analysis of the intersection between multi-sensor fusion and XAI in the context of AV systems. In this extended review study, we aim to systematically review state-of-the-art multi-sensor fusion techniques alongside emerging XAI methodologies that contribute to the development of more transparent and interpretable AV systems without compromising safety and perception accuracy. Section 2 presents an overview of the latest advancements in multi-sensor fusion techniques and provides insight into how multi-sensor fusion methodologies are used to create a unified understanding of the vehicle’s surrounding environment. In addition, this section evaluates their respective strengths and weaknesses as well as the challenges associated with real-world autonomous driving applications.

Section 3 outlines the core principles and frameworks of XAI and presents an overview of emerging XAI techniques and tools that can be adopted to enhance the interpretability, transparency, and trustworthiness of an AV system. Furthermore, this section explores the role of XAI in AVs and emphasizes the critical importance of implementing explainability into the decision-making processes and its challenges to provide clear and interpretable insights into how and why specific driving decisions are made. Lastly, Section 4 presents a summary overview of the key findings and insights presented throughout the research and highlights future research directions that could contribute to the development of more reliable, interpretable, and trustworthy autonomous driving systems.

## 2. Multi-Sensor Fusion in Autonomous Vehicles

**Figure 3:**
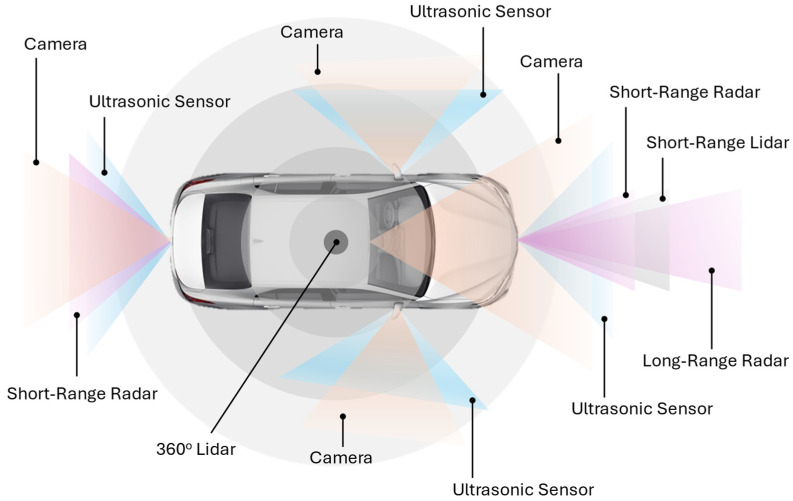
An illustrative example of a typical sensor configuration employed for environmental perception in on-road automated driving systems. It is essential to recognize that the arrangement and integration of sensor modalities can differ significantly based on operational requirements and specific applications, i.e., off-road versus on-road use cases. Other sensors, such as GPS and IMUs, are not indicated in the illustration. The image shown was redrawn and modified based on the diagram in references [39,40].

In AV systems, multi-sensor fusion serves as a cornerstone process in constructing a precise and dependable model of the driving environment. It enables the AV to interpret, predict, and respond to diverse and complex road conditions without little to no human intervention. Unlike traditional vehicles, which rely exclusively on human drivers to perceive and respond to road conditions, AV systems employ a range of sensor types, including cameras, Lidar, radar, and ultrasonic sensors, that capture unique aspects of the driving environment for safe navigations and decision-making [11]. Figure 3 below provides an illustrative example of a standard sensor configuration for environment perception in AV systems. Nevertheless, it is important to note that the arrangement and integration of various sensors can differ significantly based on the specific application scenarios and operational requirements of the AV [36,37,38,39,40].

However, each sensor type carries specific limitations that can compromise its reliability in isolation. For example, cameras deliver high-resolution images that are invaluable for capturing texture and color details and object recognition, but their effectiveness decreases in low light, glare, or adverse weather conditions. Lidar sensors generate detailed depth maps of the environment that enhance spatial awareness and enable precise three-dimensional (3D) perception. Advanced Lidar technology, such as Frequency Modulated Continuous Wave (FMCW) Lidar, extend these capabilities by providing reliable relative velocity measurements through the exploitation of the Doppler effect, which is particularly valuable for dynamic scene analysis and object tracking in real-time applications. In addition, the exploitation of Doppler effect facilitates more seamless integration and synchronization of data with radar sensors during multi-sensor fusion [41,42]. However, the performances of Lidar sensors can degrade under heavy fog or rainy weather conditions [43,44,45]. Radar sensors, on the other hand, provide reliable distance and velocity measurements without weather condition constraints, but they lack the resolution needed to capture finer details or identify static objects with precision. Lastly, ultrasonic sensors complement the perception suite in AV systems by providing short-range object detection capabilities, which are vital for close-proximity maneuvers such as parking, yet their capabilities are limited by their short operational range and they are not suitable for use in high-speed driving scenarios, where higher-resolution data and broader spatial awareness are indispensable [11,46,47].

Hence, incorporating multiple sensor data streams utilizing multi-sensor fusion techniques is important for overcoming the limitations that arise when sensors are used independently. In addition, the multi-sensor fusion process significantly enhances the overall robustness and accuracy of perception in AV systems, which is vital for their performance in dynamic, unpredictable, and safety-critical driving scenarios. Table 2 below presents a summary of the advantages and limitations associated with exteroceptive sensors—cameras, Lidar, radar, and ultrasonic sensors [48,49]. It underscores the advantages and limitations of the sensors, providing valuable insights into their performance across different operational requirements and environment or illumination conditions.

In the context of multi-sensor fusion, several distinct strategies were introduced and adopted to integrate data from multiple sensor modalities to improve the overall perception and decision-making capabilities of AV systems [57]. These strategies can be broadly categorized into three primary approaches: (a) low-level fusion, (b) mid-level fusion, and (c) high-level fusion. Each of these approaches presents a distinct technique for integrating sensor data, designed to optimize the trade-offs between data richness, real-time processing requirements, and computational efficiency. By strategically integrating data at different stages within the sensor data processing pipeline, these fusion techniques aim to address the inherent limitations and uncertainties of individual sensor modalities to create a more robust and resilient perception and navigation model in AV systems. This, in turn, allows AV systems to achieve a higher level of situational awareness, improving the reliability of decision-making and ensuring safer navigation, even in complex and challenging driving environments [11,57,58,59].

### 2.1. Multi-Sensor Fusion Approaches

#### 2.1.1. Low-Level Fusion

Low-level fusion (LLF), also known as data-level fusion or early fusion [59,60,61], represents the most granular approach to integrating sensor data in AV systems, where data from multiple sensor types is integrated at the lowest abstraction level, before any significant preprocessing, filtering, or feature extraction occurs. In essence, the LLF approach to multi-sensor fusion utilizes raw features or unprocessed sensor inputs, such as raw radar reflections, camera pixel data, or Lidar point clouds, to create a comprehensive, high-resolution representation of the driving environment. One of the key advantages of LLF approach is its capability to retain the fine-grained information captured by each individual sensor, which maximizes the amount of information available for further analysis, including small objects or minute changes in the driving scene. As a result, the LLF approach plays an essential role in enhancing the precision and reliability of object detection and environmental awareness in an AV’s perception system, specifically in dynamic or complex driving scenarios where capturing and preserving fine-grained information is critical for accurate decision-making and ensuring safe navigation [62].

In AV systems, the LLF strategy is often employed in scenarios where high precision and fine-grained detail are indispensable, especially in tasks such as object detection, classification, and tracking. For instance, a recent study by [63] demonstrated that integrating high-resolution camera images and Lidar 3D point clouds at the raw data level substantially improves the accuracy of image depth estimation. It involves projecting Lidar point clouds onto the image plane, otherwise known as sparse depth maps, and further refines into dense depth maps utilizing a depth completion method [64] to transform camera features into a bird’s-eye view (BEV) space for long-range high-definition (HD) map generation, thereby improving the precision of object detection and overall spatial awareness. In addition, the study referenced in reference [65] introduced a novel camera–radar fusion transformer framework to integrate spatial and contextual information from both the radar and camera sensors using an innovative Spatio-Contextual Fusion Transformer (SCFT) model and a Soft Polar Association (SPA) module. It leverages the complementary strengths of each sensor and the associated polar coordinates between radar points and vision-based object proposals for object detection, classification, and tracking. This approach achieved state-of-the-art performance on the nuScenes test dataset [66], outperforming other existing camera-radar fusion methods in terms of accuracy and reliability.

**Figure 4:**
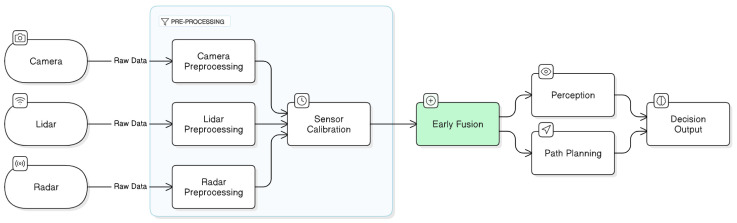
A graphical representation of the concept and architecture of the LLF strategy for multi-sensor fusion. It visualizes the step-by-step fusion processes at the high level, emphasizing on how raw sensor data streams from multiple sensor modalities are pre-processed, e.g., multi-sensor calibration, prior to being integrated into a unified dataset for further analysis. The diagram illustrated was modified and redrawn based on the depiction in references [67,68].

Figure 4 below illustrates the concept and architecture of the LLF approach to multi-sensor fusion. It visually demonstrates a high-level overview of the step-by-step fusion processes, emphasizing how raw data streams from an array of sensor modalities are pre-processed, including spatial-temporal calibration [11], prior to being integrated into a unified dataset for further perception and navigation analysis [67,68]. While LLF is advantageous in providing a comprehensive, detailed view of the surrounding environment, it is not without its challenges and drawbacks. LLF requires high computational resources and memory bandwidth to manage and process large volumes of raw data from multiple sensors simultaneously, specifically at high resolutions. It leads to increased latency and may negatively impact processing capabilities, which is not suitable in complex, dynamic environments where real-time decision-making is essential. Besides, LLF is susceptible to errors in the spatial-temporal calibration of sensors operating at different frequencies. In safety-critical AV systems, these sensor misalignments can lead to inaccuracies in detecting objects and predicting object distances and trajectories, thus compromising the reliability and safety of the AV systems. Besides, the LLF approach exhibits limited flexibility in scenarios where a sensor fails or malfunctions, as the tightly coupled architecture relies heavily on synchronized inputs from all sensors. Thus, such dependencies reduce the robustness of the system and can pose significant challenges in maintaining the operational safety of the AV system in real-world conditions [67,68,69].

#### 2.1.2. Mid-Level Fusion

In contrast to LLF, which integrates raw data to build a comprehensive and detailed representation of the surrounding driving environment, mid-level fusion (MLF) utilizes the extracted salient features from individual sensor types to construct a more refined and computationally efficient perception of the surroundings. MLF, otherwise known as feature level fusion, intermediate fusion [68], or middle-fusion [70], integrates the high-level features obtained from individual sensors, such as depth estimations from Lidar, motion trajectories from radar, object boundaries from cameras, et cetera, to develop a more abstract yet informative representation of the environment [59]. The MLF approach to multi-sensor fusion lies in its ability to balance perception accuracy with computation efficiency, especially in real-time decision-making scenarios. It offers a pragmatic solution for AV systems by optimizing the allocation of resources and reducing the computational complexity of sensor data processing while maintaining the precision of situational awareness for effective and safe navigation in dynamic, real-world driving conditions [71].

The MLF approach is often adopted to achieve a balance between high-accuracy perception and computational efficiency in real-time data processing for object detection, classification, and tracking. In their study, the authors of reference [72] introduced Contextual Fusion, an environmental-based fusion network that leverages domain-specific knowledge about the limitations of camera and Lidar sensors as well as contextual information about the environment to enhance the perception capabilities. It utilizes the MLF approach to integrate features extracted from the sensors and environmental contextual data, i.e., illumination conditions—daytime and nighttime—and rainy weather conditions, to detect objects in adverse operating conditions, achieving state-of-the-art detection performance on the nuScenes dataset [66] during nighttime. In reference [73], the scholars presented the concept of an end-to-end perception architecture that leverages the MLF strategy in its deep fusion network to create a shared representation of the surroundings. Its fusion network incorporates the features obtained from individual sensor encoders, as well as the temporal dimensions, to develop a unified latent space that is sensitive to the nuances of spatial relationships and temporal dynamics for subsequent perception tasks, including object detection, localization, and mapping. By utilizing the unified latent space, the network allows interdependent learning across various perception tasks to minimize redundant data processing, hence optimizing resource utilization and computational efficiency.

**Figure 5:**
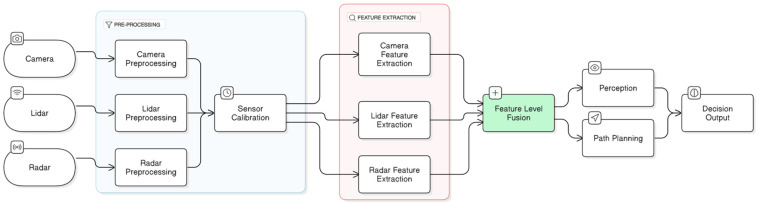
A graphical representation of the concept and architecture of MLF approach to multi-sensor fusion. It visualizes the high-level overview of the MLF processes, where features such as depth estimations and texture gradients were extracted from individual sensors prior to being integrated into a unified dataset for further perception and safe navigation processing to support accurate and safe driving tasks. The diagram shown was redrawn and modified based on the depiction in reference [68].

Figure 5 below depicts the concept and architecture of the MLF approach to multi-sensor fusion. It illustrates a high-level overview of the sequential fusion processes, emphasizing how distinct features are initially extracted from individual sensor types prior to being integrated into a shared feature space for subsequent perception and navigation analysis [67,68]. Although MLF offers significant benefits in optimizing resource utilization while maintaining high object detection accuracy, it also presents certain challenges and limitations. MLF requires robust feature extraction algorithms to accurately synthesize the relevant information from disparate sensor sources. It relies on precise feature extraction and is vulnerable to sensor failures, noise, and inconsistencies, which can lead to information loss resulting in degraded performance in critical perception tasks [59]. Additionally, MLF requires precise multi-sensor spatio-temporal calibration to ensure data consistency during the fusion process. It also requires substantial computational resources to integrate large feature subsets from multiple sensors, which can be challenging in real-time safety-critical systems due to concerns about data latency [11]. Furthermore, as noted in reference [74], the MLF strategy may not be adequate to support the realization of SAE Level 4 or 5 AVs, as it struggles to handle unexpected scenarios based on predefined feature sets and may fail to retain critical contextual information.

#### 2.1.3. High-Level Fusion

High-level fusion (HLF), also referred to as decision-level fusion or late fusion [68], represents the highest level of abstraction for integrating multi-sensor data in AV systems. In contrast to LLF and MLF, HLF incorporates individual sensor outputs or decision-making results to construct a comprehensive understanding of the environment. It focuses on integrating the final interpretations or outcomes derived from the analysis performed by individual sensors, such as location coordinates, velocity vectors, motion trajectories, predicted bounding boxes, classifications of detected objects, et cetera, to establish a reliable, unified, and accurate informed decision [70,75]. One of the key benefits of the HLF approach is its modular structure that allows seamless integration of new sensors or updates to existing multi-sensor fusion system without significant changes to the overall fusion framework. As a result, it can be easily adapted to incorporate additional sensing modalities or to accommodate multiple sensor configurations, thereby supporting the scalability of the autonomous driving system [68]. Furthermore, HLF enhances computational efficiency by focusing on the integration of high-level decisions from individual sensor modalities, which significantly reduces computational complexity compared to raw sensor data as the processed, abstracted information requires fewer resources, making it beneficial for low-latency applications in AV [76]. HLF also promotes robustness and fault tolerance due to its approach to sensor fusion, which allows the system to maintain effective operation when one or more sensors fail or provide erroneous data—no interdependence at the feature or raw data levels.

HLF approach is often adopted to optimize computational efficiency while maintaining effective decision-making capabilities and overall system performance, specifically in real-time, safety-critical applications such as autonomous driving. In their study, the authors of reference [77] introduced a multi-modal multi-class late fusion (MMLF) architecture, which integrates object-level information from various sensor modalities and quantifies the uncertainty associated with the classification results. It involves integrating bounding boxes (spatial locations of objects) from the detectors and non-zero Intersection over Union (IoU) values to obtain multi-class features for uncertainty estimation. As a result, the integration leads to improved precision and reliability in object detection, achieving substantial performance improvements on the KITTI [78] validation and test datasets. In reference [79], the researchers presented a late fusion architecture that leverages Deep Neural Network (DNN) models to detect pedestrian detection during night-time conditions by utilizing data inputs from RGB and thermal camera images. This involves integrating the outputs, i.e., bounding boxes and detection confidence scores, from individual detection models and applying a Non-Maximum Suppression (NMS) method [80] to eliminate redundant detections of the same object and refine the final detection outputs. As a result, the architecture enhances the precision and reliability of pedestrian detection in night-time conditions while ensuring an optimal balance between detection accuracy and low response time during real-time inferencing.

**Figure 6:**
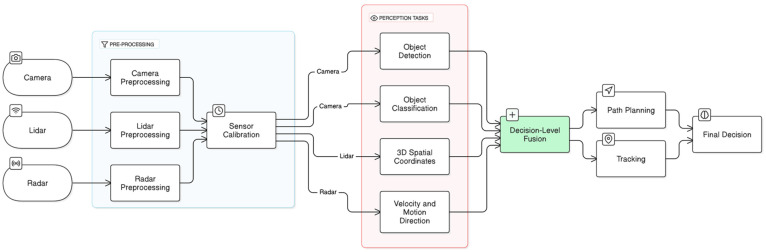
A graphical representation of the conceptual framework of the HLF approach to multi-sensor fusion. It visualizes the high-level overview of the HLF processes, emphasizing how the flow of information as data from individual sensors undergoes independent analysis before the fusion stage occurs to establish a unified informed decision. The depiction shown was adapted and redrawn based on the illustration in reference [68].

Figure 6 below demonstrates the concept of the HLF approach to multi-sensor fusion. It visualizes the high-level overview of the HFL processes, where the outputs generated by individual sensor data analysis are integrated to achieve enhanced situational awareness and reliable informed decisions in dynamic driving scenarios [67,68]. While HLF strategy is advantageous in terms of its computational efficiency and modularity, it is not without its challenges and drawbacks. One notable drawback is the potential loss of detailed contextual information that is often available in raw or feature-level data. HLF may overlook the fine-grained details that are crucial for precise decision-making, especially in dynamic and complex driving environments. The omission of these details can result in erroneous or suboptimal decisions, which can negatively impact the overall performance and safety of the autonomous driving system [70]. Additionally, the HLF approach relies significantly on the precision and reliability of each individual sensor’s interpretation of the surroundings. In other words, any inaccuracies, misclassifications, or failures in the data from a single sensor can propagate through the AV system, which can lead to misinterpretation of objects or incorrect assessments of driving conditions [59].

From a computational perspective, sensor fusion can also be categorized into (a) centralized fusion, (b) decentralized fusion, and (c) distributed fusion. Each of these categories defines the architecture and the specific locus of where the fusion process occurs within the system [81]. In centralized fusion, raw data from each individual sensor is transmitted to a central processing unit, where it is integrated to produce a cohesive and comprehensive representation of the surroundings. In other words, the central processor handles a range of critical tasks in autonomous driving, including data filtering, feature extraction, decision-making, and overseeing system control functions, to ensure safe and efficient autonomous driving. In contrast to centralized fusion, decentralized fusion distributes the fusion process across multiple local nodes, where each sensor or subsystem independently processes its data and performs local fusion or analysis before transmitting the processed results to a central unit or other nodes for further integration. In distributed fusion, the concept of decentralization is further extended to allow each sensor or node to share intermediate or partially fused results across the system without relying on a single central processing unit for final decision-making. Table 3 below highlights the advantages and drawbacks of centralized fusion, decentralized fusion, and distributed fusion [49,81,82,83].

ISO 26262, titled “Road Vehicles—Functional Safety”, is an international standard for functional safety of electrical and/or electronic systems that are installed in series-production road vehicles with a maximum gross vehicle mass up to 3500 kg. It mandates a structured development process that incorporates redundancy to minimize the risks associated with system failures or malfunctions, thereby ensuring automotive safety [84,85]. In reference [86], the authors established an architecture to ensure availability and reliability in AVs based on hardware redundancy and software redundancy to improve operational safety. From a hardware perspective, which comprises various components including communication modules, sensors, actuators, power supplies, and other critical elements, redundancy can be incorporated at multiple levels to improve system reliability and functionality. Specifically, sensor-level redundancy can be applied to ensure continuous environmental monitoring even in the event of sensor failure, which can be accomplished by deploying multiple sensors of the same or complementary types. For instance, if a camera sensor fails to deliver accurate data due to conditions such as strong sun glare, other sensors in the AV system can compensate for these limitations and ensure the continued provision of reliable environmental perception [86]. Furthermore, communication redundancy is indispensable to ensure continuous connectivity with essential internal and external networks, e.g., traffic infrastructure, other vehicles, and cloud services, in the event of network failures, malicious attacks, interference, or data corruption. It involves creating multiple communication pathways, channels, and failover mechanisms to reduce the risk of system downtime and ensure the resilient and uninterrupted flow of key information for real-time decision-making processes [87].

From a software perspective, redundancy can be deployed to mitigate potential failures that may arise from both hardware and software components, such as software malfunctions, algorithmic errors, or system crashes. At the software level, redundancy plays an imperative role in enhancing system resilience against software malfunctions by offering backup mechanisms that ensure the uninterrupted and reliable execution of key data processing tasks and control algorithms. It safeguards the overall functionality of the system and maintains its operational continuity in the event of unexpected disruptions, failures, or errors, thereby ensuring the reliability and robustness of the autonomous system in dynamic and fault-prone environments [86]. For instance, data redundancy can be utilized to provide backup information in scenarios where a particular data source becomes inaccessible or compromised. By adopting redundant data sources, the system can cross-verify sensor outputs and ensure data accuracy, thereby enhancing confidence and reliability in the fused data [88]. At the software level, processing redundancy can also be utilized to maintain robustness in data processing, particularly in scenarios where a specific algorithm or processor fails or generates erroneous outputs. It involves utilizing multiple processing units or parallel algorithms that independently handle sensor data, with their outputs cross-verified to improve confidence in the integrated outputs [89,90]. However, implementing and maintaining redundant hardware and software components increases the complexity of the system architecture, presenting significant challenges in system design, testing and verification, and maintenance processes. Besides, the inclusion of redundant components increases overall cost and requires precise synchronization, efficient resource management, and the utilization of robust fault management strategies [85]. For a comprehensive discussion of the different redundancy strategies applicable to safety-critical AV systems, readers are advised to refer to references [91,92,93,94,95].

In summary, by strategically integrating sensor data at different stages of the multi-sensor processing pipeline, these multi-sensor fusion approaches aim to leverage the complementary strengths of diverse sensors and the architectural designs of the autonomous driving systems. As discussed, multi-sensor fusion can occur at both the abstraction level, i.e., HLF, MLF, and LLF, and computational level, i.e., centralized fusion, decentralized fusion, and distributed fusion. On the one hand, the sensor fusion approaches at the abstraction level dictate the timing of when data from individual sensors are integrated. In other words, it addresses the question of “when should the multi-sensor fusion occur?”. On the other hand, the fusion approaches at the computational level emphasis on the location in which the fusion process occurs to optimize system performance. In essence, it addresses the question of “where should the multi-sensor fusion occur?”. Nonetheless, it is vital for readers to learn that sensor fusion can also occur at the competition level, which addresses the question of “what should the fusion do?” [81,82,96] (detailed discussion of the fusion approaches at the competition level, i.e., competitive fusion, coordinated fusion, and complementary fusion, is beyond the scope of this manuscript). Ultimately, selecting the most suitable sensor fusion approach depends on the specific use cases and requirements of the AV systems, including scalability, computational resources, fault tolerance, real-time performance, and system design.

Nonetheless, recent advancements in ML and DL technologies have significantly accelerated the development of end-to-end solutions in autonomous systems. Unlike traditional architectural paradigms, which depend on modular pipelines consisting of distinct subtasks including perception, localization and mapping, decision-making and planning, and control, end-to-end solutions incorporate these components into a unified and interconnected structure, as exemplified in reference [97] (Figure 5). In essence, the end-to-end architectural solution offers a more streamlined and efficient technique for multi-sensor fusion by leveraging advanced DL methods to continuously train, learn, analyze, and optimize the complex interrelationships among various environmental features within large-scale raw sensor datasets. For instance, NVIDIA introduced an innovative end-to-end architectural paradigm known as Hydra-MDP, which represents a significant achievement in generalization across diverse driving environments and conditions. Hydra-MDP uses a teacher–student model and leverages multiple teacher networks to learn how environmental factors impact planning strategies. Additionally, it integrates knowledge from human driving behaviors and rule-based planners in a scalable manner to enable end-to-end planning predictions. As a result, this method has secured first place in the CVPR End-to-End Driving at Scale challenge [98,99]. Despite the benefits of end-to-end approaches in autonomous systems, significant challenges remain in ensuring the reliability, safety, and explainability of these systems due to the lack of clear insights into their underlying decision-making processes, particularly when DL black-box models are utilized [97].

### 2.2. Fusion Techniques and Algorithms

In AVs, multi-sensor fusion methods and algorithms serve as the cornerstone for building robust and precise systems that enable reliable perception, accurate localization, and efficient navigation. They support the integration of data from various sensor types such as GPS, camera, Lidar, and radar sensors to construct a more comprehensive understanding of the surroundings, thereby enhancing situational awareness in the highly dynamic and complex driving environment. Over the years, the sensor fusion techniques and algorithms have been studied significantly and become well established in the literature [59,60,68,100,101,102,103,104,105,106,107,108]. Fusion techniques and algorithms can be classified into (a) traditional approaches and (b) advanced approaches. In traditional approaches, the algorithm utilizes well-established mathematical frameworks, such as deterministic rules, probabilistic theories, and optimization-based criteria, to combine data from multiple sensors. It offers robust, efficient, and interpretable solutions to multi-sensor fusion, specifically in scenarios where the systems require transparency in their decision-making processes and have limited computational resources. Nonetheless, traditional approaches can pose a challenge in nonlinear, highly dynamic, and unstructured environments. Their reliance on predefined models or assumptions about the data distribution may result in suboptimal performance when the assumptions are inaccurate or violated [100].

Conversely, algorithms in advanced approaches leverage complex DL techniques to process, analyze, and integrate data from various sensors. It represents a significant shift towards data-driven methodologies as it employs a multi-layered structure of algorithms (also known as deep neural networks [109,110]) and big data to learn the complex representations, nonlinear relationships, and intricate patterns between multiple sensor inputs for multi-sensor fusion. Essentially, these algorithms are designed to adapt to complex, high-dimensional, and unstructured data, such as camera images, which enables the algorithms to generalize effectively across diverse and dynamic real-world driving environments. As a result, the algorithms provide enhanced perception and navigation capabilities, ensuring reliable performance in challenging and dynamic driving conditions. Nevertheless, as algorithms in advanced approaches continue to advance, their lack of interpretability presents significant challenges in ensuring safety, trust, accountability, and transparency in their decision-making processes, particularly in critical applications such as AVs. Besides, DL techniques are computationally complex due to their intricate underlying architecture, which can lead to increased latency and resource consumption [11,100,111].

**Figure 7:**
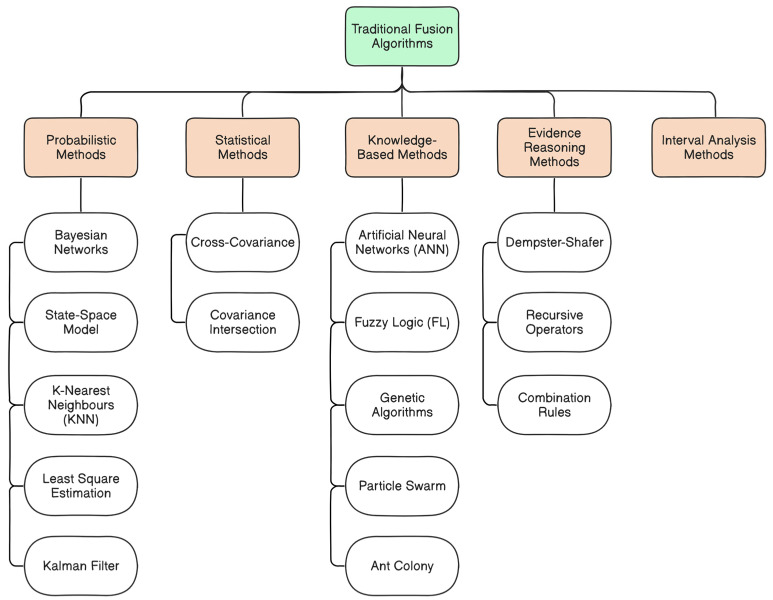
A graphical summary of the traditional fusion methodologies and their associated techniques and algorithms. It highlights the various algorithms used within different paradigms such as probabilistic method, statistical method, knowledge-based method, evidence reasoning method, and interval analysis method. The diagram shown was redrawn based on the illustration in reference [100].

**Figure 8:**
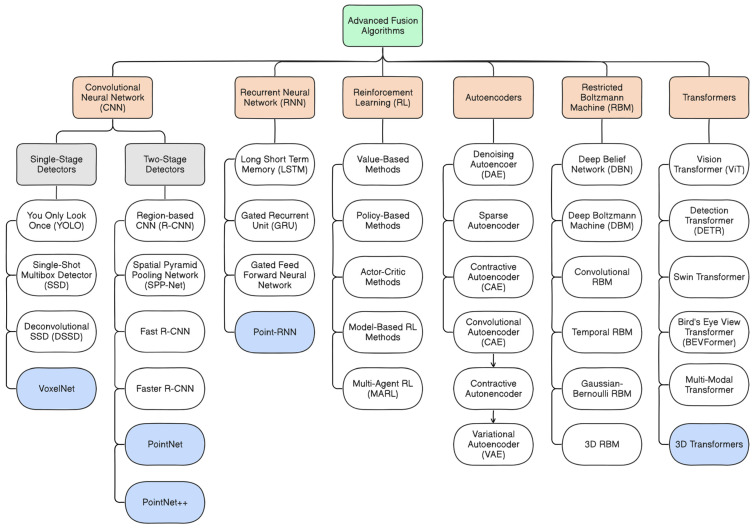
A graphical overview of the advanced fusion methodologies and their associated techniques and algorithms. It emphasizes the various DL algorithms applied within different paradigms for perception, localization, and mapping systems in AV applications. The figure shown was redrawn and adapted based on the depiction in references [59,68,100,103,114,115,116,117,118,119,120,121,122] to include state-of-the-art algorithms, and the algorithms highlighted in “blue” represent those specifically utilized for perception tasks involving 3D point clouds.

Figure 7 and Figure 8 below demonstrate the traditional and advanced approaches, respectively, highlighting examples of techniques and algorithms that are commonly used in AV systems for tasks such as object detection, localization, and navigation. Figure 7 exemplifies the traditional fusion algorithms, which include well-established techniques that rely on mathematical models, statistical approaches, knowledge-based theory, and probabilistic frameworks. These techniques are often adopted in scenarios where the dynamics of a system are well understood and the noise characteristics are predictable [100]. In reference [112], the scholar utilized the Unscented Kalman Filter (UKF) algorithm, an adaptation of the Kalman Filter (KF) algorithm for nonlinear state estimation [113], to incorporate GNSS absolute positioning values and real-time IMU input data. It addresses the potential drift inherent in IMU data during sensor fusion processes, ensuring accurate and reliable estimates of the vehicle’s position and orientation and ultimately improving the robustness and precision of the navigation system in AVs. Figure 8 depicts the advanced fusion algorithms, which leverage modern DL approaches such as Convolutional Neural Networks (CNN), Recurrent Neural Networks (RNN), Restricted Boltzmann Machine (RBM), Transformers, Reinforcement Learning (RL), and Autoencoders [59,68,103,114,115,116,117,118,119,120,121,122]. These techniques are effective in processing complex, high-dimensional input data and are designed to adapt to the dynamic and unpredictable characteristics of real-time driving environments. For example, the scholar in reference [123] contributed to a novel multi-object tracking system that utilizes three trained Long Short Term Memory (LSTM) models to perform data association, tracking updates, and object position estimation. LSTM model is an RNN-based technique that is designed to capture long-term dependencies in sequential data, which is ideal for tasks like time-series prediction of an object trajectory or vehicle motion prediction [124].

In complex applications like autonomous driving systems, traditional and advanced fusion algorithms are commonly utilized in tandem to leverage the strengths of each approach, also known as the hybrid approach [125,126]. This synergistic integration is critical for achieving optimal performance in diverse tasks, such as environmental perception and motion trajectory estimation, where the robustness and efficiency of traditional methods complement the adaptability and learning capabilities of advanced DL algorithms. In reference [127], the authors proposed a hybrid approach to develop a parameter-free state estimation framework for GPS-based maneuvering-target tracking and localization in AV applications. It features a parameter learning module that integrates a transformer encoder architecture with an LSTM network to effectively capture the motion characteristics of the system from offline state measurement data. In addition, the framework incorporates the Expectation-Maximization (EM) algorithm, which is a well-established statistical approach for parameter estimation in probabilistic models [128]. The EM algorithm estimates the measurement and dynamic characteristics of moving targets in real time and refines the system parameters based on the outputs of the learning module. Lastly, a KF algorithm is used to deliver precise statement estimations, thereby enhancing the accuracy of trajectory tracking predictions. This synergistic integration of traditional algorithms and advanced learning techniques provides a robust solution to estimate state and track trajectory of maneuvering targets in real time. Hence, it effectively mitigates the impact of sensor noise, e.g., Doppler shift, occlusion, and flicker, and eliminates the need to explicitly model the complex dynamics and measurement characteristics of the system.

In reference [129], the authors introduced YOLO-ACN, a novel and efficient detection framework specifically developed to improve detection precision and overcome the challenges of detecting small targets and occluded objects within complex environments. It includes a lightweight feature extraction network with an attention mechanism, built upon the architecture of the You Only Look Once (YOLO) neural network, particularly YOLOv3 [130], to improve focus on small target detection. YOLO is a single-stage detector that simultaneously predicts multiple bounding boxes (detected objects) and class probabilities on an image in real time [131]. In addition, the network features a modified variant of the NMS classical algorithm, referred to as Soft-NMS, within its post-processing phase to eliminate redundant bounding boxes while reducing the likelihood of discarding occluded objects, especially in densely populated environments. Unlike traditional NMS, which eliminates overlapping bounding boxes that exceed the predefined IoU threshold, Soft-NMS retains overlapping boxes with adjusted confidence scores, thereby improving detection performance in complex scenarios [132,133]. As a result, this synergistic integration has significantly enhanced detection performance and robustness, particularly in recognizing small targets and occluded objects within complex environments, such as urban areas with high pedestrian density.

Ultimately, the selection of the most suitable techniques for the hybrid approach depends on the specific requirements and use cases of the intended application. In complex and dynamic scenarios, leveraging a combination of traditional and advanced algorithms has become a preferred strategy to capitalize on their complementary strengths. This combination not only enhances overall performance but also improves the precision and reliability of the system, ensuring that it is optimized to address the distinct challenges associated with each driving task. Table 4 below provides an overview of the advantages and weaknesses of both traditional and advanced learning algorithms utilized in multi-sensor fusion systems for AV applications, such as UKF, Particle Filter (PF), YOLO, Dempster–Shafer Theory (DST), PointNet, and Faster R-CNN [11,100,134,135,136,137,138,139,140,141,142,143,144,145,146,147,148,149,150,151,152]. In addition, this table focuses on their applications in dynamic driving tasks, such as object detection, tracking, and localization and mapping, which are essential for the safe and efficient operation of autonomous driving in complex and dynamic driving settings. For a comprehensive discussion of traditional and advanced learning methods for object detection in 3D point cloud data (out of the scope of this manuscript), readers are recommended to refer to references [68,103,105,106,108,118,122,153,154,155].

### 2.3. Challenges in Multi-Sensor Fusion

In AVs, integrating multiple sensor data, otherwise known as multi-sensor fusion, is a cornerstone for implementing precise and robust systems capable of achieving the high levels of perception, localization, and mapping essential for autonomous operations. By synergistically integrating information from complementary sensor modalities, multi-sensor fusion allows AVs to construct a comprehensive and dynamic understanding of their environment. In addition, by leveraging the unique strengths of various sensors and traditional and advanced fusion algorithms, multi-sensor fusion significantly enhances the capability of AVs to detect obstacles, interpret traffic patterns, and navigate effectively through complex and unpredictable driving environment. Nonetheless, while multi-sensor fusion has revolutionized the capability of AVs to interact effectively with their surroundings, it also introduces several critical technical, operational, and interpretability challenges that need to be addressed for the successful deployment of reliable, safe, scalable, and interpretable (transparent) autonomous systems in real-world applications.

One of the primary challenges is sensor noise, which refers to inaccuracies, inconsistencies, or irrelevant data introduced by individual sensors due to a combination of external interference, hardware limitations, and environmental conditions, such as rain, snow, or dense fog. In reference [67], the authors presented a comprehensive overview of the challenges associated with radar technologies in autonomous driving systems. A major issue identified is the occurrence of spurious observations, also known as clutter, which arises due to multiple reflections off surfaces in the surroundings, a phenomenon commonly known as multipath. In some cases, such clutter can be difficult to distinguish from real detections, leading to false positive detections in learned radar-based detection models. This, in turn, can significantly undermine the overall system performance and the ability to make precise, reliable, and trustworthy decisions. In our previous exploratory research [11] (Figure 4), we observed multiple instances of false-positive and inconsistent detections within the off-road testing environment, which includes metal objects with corrugated surfaces, traffic cones, and guardrails. These issues were caused by multipath propagation, which distorts sensor signals and leads to inaccurate and unreliable detections in complex environments [158]. A study in reference [159] showed that Lidar sensors can generate false-positive detections in rainy weather due to reflections from raindrops, and wet surfaces may cause laser beams to scatter, resulting in artifacts such as mirrored objects appearing below the actual ground surface. Therefore, these factors can undermine the accuracy and reliability of the sensor outputs, posing significant challenges for ensuring the reliability and precision of autonomous driving operations.

In addition, the heterogeneity of sensor modalities and the ensuing system complexity represent another major challenge in multi-sensor fusion. AVs are generally equipped with a diverse set of sensor types, including cameras, Lidar, radar, ultrasonic sensor, and GPS, each with distinct operational attributes that contribute to their strengths and weaknesses. For example, radar is resilient in poor weather but offers lower spatial resolution; Lidar offers high-resolution depth information but is computationally intensive; and cameras capture rich visual detail but are sensitive to lighting and weather conditions. Nonetheless, integrating these diverse sensor types introduces significant complexity in algorithmic design and computational processing. Doing so requires sophisticated and innovative fusion algorithms that can handle differences in sensor data format, resolution, and spatial-temporal synchronization [11] while maintaining the overall AV system performance and reliability. Moreover, the complexity of the fusion systems escalates as additional sensors are incorporated to enhance the robustness of perception and support real-time decision-making. This results in the generation of big data, imposing significant demands on computational resources and necessitating innovative real-time processing capabilities to maintain timely and accurate responses. Furthermore, it also intensifies the difficulties associated with testing and validation as rigorous evaluations across varying driving scenarios and environmental conditions are essential to minimize failure risks and ensure dependable and safe operation in real-world contexts [160,161].

In AVs, the volume of data generated by multi-sensor fusion systems is significantly extensive, highlighting the complexity and sophistication of the sensor suites employed to perceive and navigate the environment. The continuous operation of these sensors generates high-dimensional, multi-modal data streams, with throughput often reaching multiple gigabytes per second or even terabytes per hour, depending on system configuration (how many sensors are integrated into the system), sensor resolution, refresh rates, and operating conditions [162,163]. This immense data volume is essential for robust perception, localization, and decision-making, but it introduces significant challenges in implementing low-latency data processing pipelines and optimizing the utilization of computational resources. In the event of delays or latency within the data processing pipeline, the AV may fail to respond to dynamic changes in its surroundings, such as unforeseen objects or pedestrians entering the roadway [164]. Besides, the limitations of computational resources in the embedded systems that are often utilized in AVs require deliberate trade-offs between accuracy and computational efficiency, requiring the optimization of complex fusion algorithms to operate within hardware constraints. Moreover, safety-critical autonomous systems require multi-sensor output verification and cross-validation to address the potential risks of sensor noise, malfunction, or environmental interference, thus posing significant challenges in its computational load [165]. Hence, addressing these challenges necessitates innovative approaches, such as leveraging parallel processing, hardware accelerators, e.g., Graphics Processing Units (GPUs) or Tensor Processing Units (TPUs), and optimized fusion frameworks [166,167,168].

In addition, multi-sensor fusion systems in AVs are susceptible to malicious attacks, which pose significant risks to the integrity and reliability of their autonomous operation. AVs rely on seamless integration of multiple sensor modalities but are vulnerable to different forms of adversarial interference, such as spoofing, jamming, and signal manipulation. For example, attackers may broadcast incorrect yet plausible GPS signals to mislead the AV about its true location, leading to navigation inaccuracies [169]. Similarly, adversaries exploit the vulnerabilities of deep neural networks and introduce subtle perturbations to images that are often imperceptible to the human eye, otherwise known as adversarial images. This causes the trained model to produce erroneous predictions or classifications [170]. Moreover, attackers may target the underlying software or communication infrastructure of the multi-sensor fusion system through cyberattacks to overload the system, disrupt data transmission, or manipulate sensor inputs. Thus, these attacks compromise the robustness and reliability of decision-making processes and endanger its overall safety during autonomous operations [171]. In recent years, the Zero Trust framework has emerged as a key approach in the design and implementation of multi-sensor fusion systems in AVs. It challenges the traditional assumption of inherent trust within the ecosystem and operates under the core principle that no component or node in the autonomous system should be automatically trusted [172,173]. For a comprehensive exploration of the different attack models and their associated defense strategies (out of the scope of this manuscript), readers are encouraged to refer to the research established in references [94,170,171,172,174,175,176,177,178].

In complex fusion algorithms, the lack of interpretability and explainability presents significant challenges in ensuring transparency and accountability in autonomous operations. One crucial aspect of this challenge is the necessity to provide clear and comprehensible explanations to stakeholders regarding the decisions and actions made by the autonomous system. For example, end-users often require comprehensible explanations to foster trust and confidence in the reliability of autonomous driving technologies, particularly in safety-critical applications such as AVs. Similarly, regulatory authorities seek comprehensive insights into the decision-making processes to evaluate compliance with well-established safety protocols, legal standards, and ethical guidelines [179]. Additionally, the necessity for explainability is critical for fostering user acceptance of autonomous driving technologies. A lack of clarity in explaining the rationale behind specific actions taken by autonomous systems, especially in situations involving errors or unanticipated outcomes, can significantly undermine user trust and hinder the acceptance of autonomous driving technologies [180,181,182]. Consequently, overcoming these challenges necessitates a focused effort to design and implement multi-sensor fusion methods and models that strike a balance between complexity and transparency by leveraging XAI techniques to provide valuable insights into how inputs from various sensors are processed and integrated. By enhancing the transparency of decision-making processes, developers can facilitate regulatory approval, enhance confidence and trust among stakeholders, and ensure that autonomous driving systems are reliable and accountable in real-world applications.

## 3. Explainable Artificial Intelligence (XAI)

**Figure 9:**
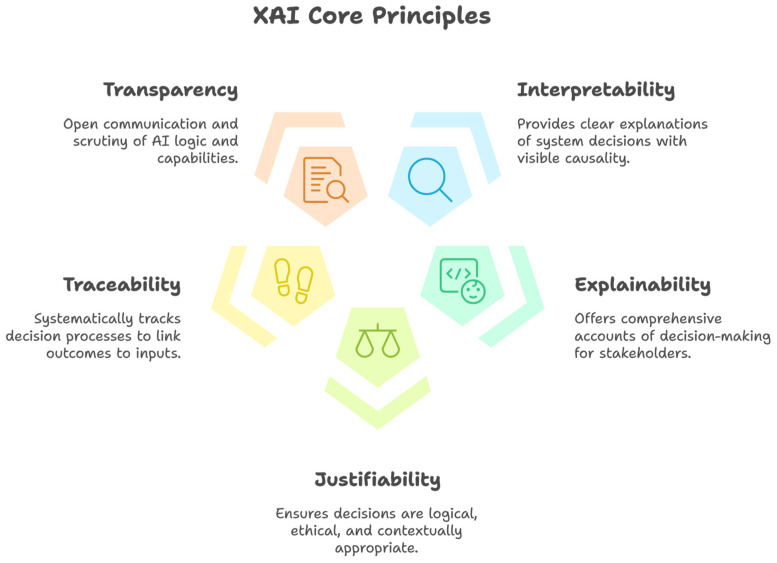
A visual depiction illustrating the five core principles of XAI: interpretability, explainability, justifiability, traceability, and transparency [186,187]. The diagram shown was generated using Napkin AI, an editing platform that transforms text into visual content [189].

XAI, or Explainable Artificial Intelligence, is a specialized domain within the broader discipline of AI that focuses on designing and developing techniques and models that are interpretable and comprehensible to all stakeholders. These stakeholders include, but are not limited to, (a) researchers and academics aiming to advance the field through theoretical and applied insights; (b) developers and engineers responsible for developing and maintaining autonomous systems; (c) end-users and consumers who interact with autonomous systems; (d) regulators and policymakers aiming to ensure compliance with established standards and safety requirements; and (e) business leaders and industry professionals focused on utilizing AI to drive commercial and operational success [183,184,185]. XAI is imperative in enhancing transparency, trust, accountability, and safety, particularly in safety-critical applications such as autonomous driving. It emphasizes five core principles that serve as foundational pillars, ensuring that such systems conform to transparency, accountability, and user trust standards while achieving their intended functionalities. XAI principles include interpretability, explainability, justifiability, traceability, and transparency, as exemplified in Figure 9 below [186,187]. It is important for readers to learn that additional XAI principles can encompass fairness, robustness, satisfaction, stability, and responsibility [188] (a comprehensive exploration of these principles is beyond the scope of this manuscript).

- Interpretability. This is defined as the ability to explain or to provide clear and comprehensible explanations of the actions and decisions made by the autonomous driving system to relevant stakeholders. It is often thought that interpretable systems are more suitable for safety-critical applications, as such systems provide a clear and observable chain of casualties that explains the decision-making processes [190].
- Explainability. This is associated with the concept of explanation as a means of providing an interface between humans and a decision-making system that is both an accurate representation of the decision-making process and comprehensible to stakeholders [191]. In essence, explainable systems can provide a clear and detailed account of how and why the decision was made.
- Justifiability. This signifies the capability of an artificial intelligence (AI) system to provide logical, ethical, and contextually appropriate reasons for its decisions (outcome) and ensuring alignment with ethical guidelines, user trusts, and accountability [192]. In essence, justifiability ensures that the decisions made by AI are justifiable and reasonable based on the given data and context. Several approaches can be used to achieve justifiability, including utilizing interpretable models, incorporating post hoc explanation tools, and involving human experts to review and validate AI decisions [192].
- Traceability. This refers to the systematic tracking and documentation of the entire decision-making process of an AI-driven system, ensuring that each action is traceable to its corresponding inputs, processing steps, reasoning, and outcomes. As a result, any anomalies or errors can be precisely identified and addressed, which is particularly essential in critical situations such as collisions or near-miss events.
- Transparency. This involves designing and developing an AI system where the underlying logic, rules, and algorithms governing the decision-making process can be scrutinized and comprehended by all stakeholders. It also involves open and clear communication with stakeholders about the decision-making criteria, functions, capabilities, and limitations of an AI system, e.g., an autonomous driving system.

**Figure 10:**
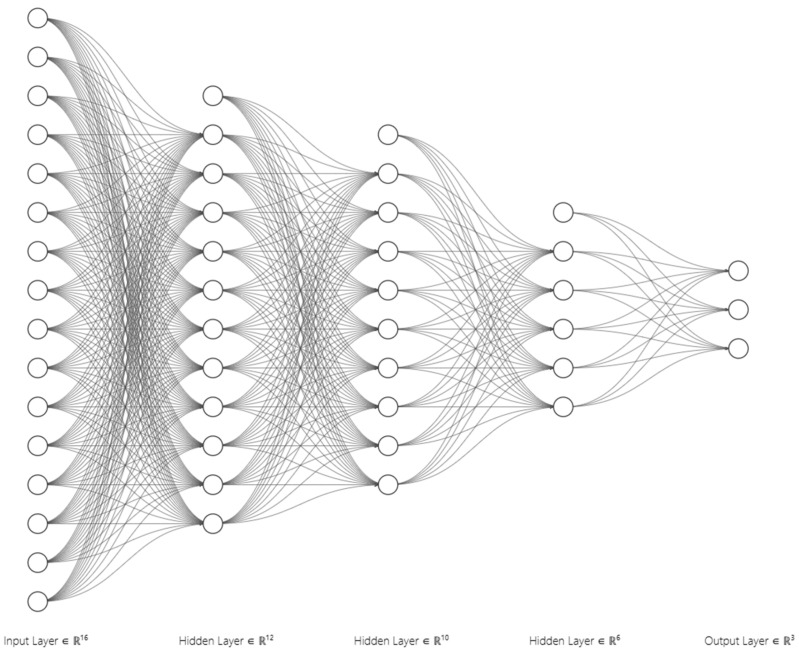
A visual representation of a Deep Neural Network (DNN) model. It shows the underlying architecture of a DNN model, which encompasses an input layer, multiple hidden layers, extensive parameterization, non-linear activation functions, an output layer, et cetera. The illustration shown was generated using the open-source NN-SVG visualization tool [195].

The rapid evolution of ML and DL techniques and algorithms has driven substantial advancements in cutting-edge autonomous applications, such as self-driving vehicles and humanoid robots [193,194]. These advancements underscore the transformative potential of ML and DL technologies in creating systems capable of performing highly sophisticated tasks, such as autonomous driving, with unparalleled precision and efficiency. However, the growing complexity and sophistication of the underlying algorithms pose significant challenges in ensuring transparency and interpretability within complex autonomous systems. In other words, the internal mechanisms of modern ML and DL models, particularly large-scale neural networks, or DNNs, and ensemble methods, are characterized by their opaque nature. Their underlying structures, i.e., multiple hidden layers and extensive parameterization, depicted in Figure 10 below [195], reflect the difficulties stakeholders encounter in comprehending the internal workings and decision-making processes of these models, resulting in their classification as black-box models or systems [196]. Furthermore, the black-box nature of DNN models introduces additional risks, including the potential propagation of biases and the complexities in diagnosing errors or unintended outcomes. In DNN models, the propagation of biases refers to the amplification or continuation of pre-existing biases embedded in the training data or unintentionally introduced during the design and implementation phases of the DNN models. This issue often arises from imbalances in training datasets, e.g., underrepresentation of specific scenarios, demographic groups, or weather conditions, as well as from implicit assumptions and inconsistencies in labeling practices and feature selection [197]. For example, underlying biases in perception algorithms used to detect objects and interpret road signs may lead to disastrous outcomes. As a result, developers use post hoc analysis techniques to elucidate the decision-making processes of black-box models. However, such methods can be resource-intensive, time-consuming, and may not always yield definitive explanations, especially when the sources of biases are deeply embedded in complex data or algorithmic structures [188,198,199,200,201].

In contrast to the black-box model, which operates an opaque system with decision-making processes that are difficult to understand, the white-box model provides enhanced transparency and offers greater insight into its internal mechanisms. It emphasizes utilizing simple and self-explanatory methods, where the decision-making processes are comprehensible and transparent to human stakeholders. A white-box model is designed with simpler underlying structures and often adopts linear or rule-based traditional algorithms, such as Decision Trees, K-Nearest Neighbors (KNN), Linear Regression, et cetera, which explicitly outline the relationship between inputs and outputs. In linear models, the predicted result can be mathematically expressed as a weighted sum of all its feature inputs, where each feature contributes to the final decision based on its assigned weight [184]. As a result, the white-box model allows a clear and direct understanding and explanation of the decision-making processes. In autonomous driving vehicles, the decision made to decelerate in response to pedestrians crossing the road can be traced and explained through a white-box model. It would generate an audit trail that outlines the rationale behind the action, including factors such as the detection of the pedestrian’s location, vehicle’s proximity to the pedestrian, and the calculated necessity to decelerate to avoid a potential collision [202]. However, the simplicity and interpretability of white-box models may struggle to attain the same level of predictive accuracy required for handling complex and dynamic real-world autonomous driving tasks, such as object detection. In addition, white-box models are often limited in their ability to effectively handle intricate and unseen scenarios, such as identifying subtle road hazards or reacting to unpredictable driver behavior [184,187,203].

In reference [186] (Figure 3), the authors presented a comprehensive discussion of the various levels of transparency that represent distinct aspects of interpretability and understanding in ML models. It comprises three distinct levels of transparency: (a) simulatability, (b) decomposability, and (c) algorithmic transparency, which serve as quintessence frameworks for understanding how the internal mechanisms of ML models can be made explainable and accessible to human stakeholders. Within transparency, there are the following levels:

- Simulatability denotes the ability to simulate the behavior of an ML model through interactive experimentation or human understanding. It enables users to replicate or anticipate the decisions made without necessitating in-depth technical knowledge of its underlying mechanisms or internal architecture. In this aspect, a model is considered simulatable if it can be effectively presented to stakeholders utilizing text, visualizations, or other accessible representations. Furthermore, a simulatable model enables users to reasonably anticipate its outputs based on a given set of inputs, fostering a more intuitive grasp of its decision-making processes [204].
- Decomposability refers to the ability to disaggregate an ML model into smaller and interpretable components, such as inputs, parameters, and computations. In essence, decomposability signifies the capability to explain the functioning of a model by examining its constituent elements, providing clarity about how specific inputs influence the outputs, how parameters are optimized, and how intermediate calculations are carried out to reach a final decision. For example, decomposability enables engineers to isolate and explain the contribution of individual subcomponents in autonomous driving, including object detection, trajectory planning, and control systems, which is critical for technical debugging, model refinement, and ensuring compliance with legal and ethical standards. However, in practice, achieving decomposability in intricate ML models, such as DNNs, can be challenging due to their non-linear relationships and the distributed nature of their data representations [186,205].
- Algorithmic transparency, as the name suggests, pertains to the extent to which the internal workings and decision-making processes of an algorithm can be clearly understood, elucidated, and scrutinized. In essence, it emphasizes the visibility of how an algorithm operates, from its initial design through to its decision outputs. In practical terms, algorithmic transparency ensures that the reasoning behind the algorithm decisions can be traced back to its underlying mathematical or computational principles, which are indispensable in identifying and rectifying potential biases, addressing embedded biases, and uncovering unintended behaviors that could compromise the precision and integrity of an ML system. In autonomous driving, understanding the decision-making processes of algorithms, such as how a vehicle decides when to stop or how it identifies and avoids obstacles, is vital in ensuring safety and adherence to regulatory standards. However, the main limitation of algorithmically transparent models is that these models must be fully accessible for analysis using mathematical methods, which is challenging for deep architectures due to the opaque nature of their loss landscapes (multiple interconnected hidden layers) [186,206,207,208,209].

The advancement of AI models (ML and DL models) has significantly amplified the need for explainability and interpretability, particularly in safety-critical domains such as autonomous driving. In these domains, it is imperative for AI systems to not only demonstrate high predictive accuracy but also deliver transparent and comprehensible explanations for their decisions to ensure safety, reliability, and adherence to regulatory and ethical guidelines. In XAI, the distinctions between black-box and white-box models underscores a fundamental trade-off in AI models development, i.e., achieving an optimal balance between interpretability and predictive performance. As discussed, black-box models are known for their ability to process complex scenarios with high accuracy but often lack transparency in understanding the underlying processes behind their decision-making. In contrast, white-box models emphasize interpretability and explainability, offering clear and understandable decision-making processes, but may face limitations in managing complex tasks.

However, both paradigms play a pivotal role in addressing the interpretability challenges inherent in cutting-edge, sophisticated AI models, significantly contributing to enhanced accountability and transparency in ML and DL technologies. Besides, both paradigms are instrumental in fostering trust among human stakeholders, which is critical in ensuring the responsible and ethical implementation of autonomous systems within real-world environments. Therefore, addressing interpretability and explainability challenges in autonomous systems has become a primary focus within XAI research, which seeks to develop tools and techniques that can elucidate the decision-making processes of opaque systems and provide human stakeholders with actionable insights into their operations.

### 3.1. XAI Strategies and Techniques

XAI is an emerging field of research that aims to provide clear, comprehensible, and human-centered explanations for the decisions generated by AI systems. Recent research has investigated several strategies and methodologies designed to elucidate the decision-making processes of intricate and opaque black-box models. XAI methods can be categorized into three main categories: (a) explanation-level, (b) implementation-level, and (c) model dependency [210]. Such categories offer a systematic framework for understanding the diverse approaches designed to enhance the interpretability and explainability of sophisticated ML and DL systems, especially in contexts where transparency is imperative. It enables researchers and practitioners to select appropriate methods or strategies tailored to specific applications and requirements.

Explanation-level refers to the scope and depth of insights delivered, addressing either the overarching behavior of the model or the rationale behind specific individual instances. This concept is subdivided into (a) global explanations and (b) local explanations. In global explanations, the emphasis is on providing a detailed overview of the model’s decision-making processes (at the macro-level). In essence, this approach delivers a holistic understanding of the model’s behavior and how it operates across different inputs and conditions. In turn, it enhances the interpretability of the model, offering insights into its underlying operational structure and the factors that influence its overall performance during the decision-making processes [210]. The Generalized Additive Model (GAM) is among the XAI methodologies that provide insights into a model’s decision-making process at a global level [211]. GAM is a statistical modeling method designed to capture and analyze non-linear relationships between dependent and independent variables utilizing smooth functions to model the effects of each predictor [212]. For instance, the research shown in reference [213] utilized the GAM method to examine the relationships between kinematic variables of vehicles, such as position, velocity, and acceleration, during overtaking maneuvers. In contrast, local explanations aim to elucidate the rationale underlying specific predictions made by the model for individual instances. It is particularly valuable in situations where understanding individual predictions is important, such as analyzing specific driving scenarios in AVs. Therefore, this approach fosters trust in high-stakes autonomous systems, ensuring safety and accountability [179,210]. Grad-CAM or Gradient-weighted Class Activation Mapping is one of the prominent XAI techniques designed to interpret the decision-making process of AI models at a local level. It is often adopted to visualize and elucidate localized decisions made by CNN-based models, particularly in image recognition and classification tasks [214]. For instance, the authors of reference [199] adopted the Grad-CAM method to analyze DL detection models by generating heatmaps that visually explain the road semantic segmentation outputs, thereby providing a comprehensive understanding of the relevance of their outcomes. Nevertheless, Grad-CAM may generate heatmaps that highlight regions unrelated to the detected objects in detection tasks, as its approach prioritizes feature importance without accounting for spatial sensitivity [215].

**Figure 11:**
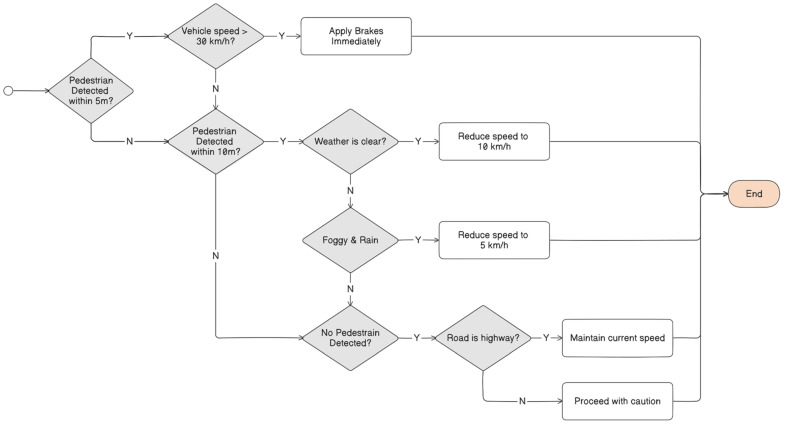
A graphical representation of the Bayesian Rule Lists (BRL) technique in elucidating the decision-making process for pedestrian crossing detection. The BRL rules shown in the illustration are derived in a preliminary manner based on our previous experimental analyses and discussions shown in reference [11]. Rule 1: If the pedestrian is detected within 5 m and the vehicle speed is greater than 30 km/h, then apply brakes immediately. Rule 2: Else if the pedestrian is detected within 10 m and the weather is clear, then reduce speed to 10 km/h. Rule 3: Else if the pedestrian is detected within 10 m and the weather is foggy or rain, then reduce speed to 5 km/h. Rule 4: Else if no pedestrian is detected and the road is highway, then maintain current speed. Rule 5: Else, proceed with caution.

Implementation-level refers to the stage at which interpretability and explainability are incorporated into AI models, focusing on when and how these aspects are integrated into the design and implementation of these models. This concept can be subdivided into (a) ante hoc explanations and (b) post hoc explanations. Ante hoc explanation, also known as intrinsic explanation or pre-hoc explanation, refers to the interpretability mechanism that is inherently integrated into the design of the model during its development phase. Such explanations are designed to embed transparency and understandability into the model’s decision-making processes from the outset, ensuring that its operation remains explainable and transparent from the initial stage [210,216]. Bayesian Rule Lists (BRL) represent a prominent example of an ante hoc explanations method. It leverages Bayesian principles to achieve an optimal balance between simplicity and predictive performance. BRL operates by composing probabilistic models that derive decision rules (IF-THEN rules) based on observed data, with a focus on selecting rules that jointly maximize the posterior probability of class labels. Therefore, BRL ensures that the resulting rule lists remain explainable and grounded in a robust statistical framework [200,217]. Figure 11 below depicts an example of how BRL can be used to explain the pedestrian crossing detection. In this instance, the model derives IF-THEN rule lists based on the input features, such as vehicle speed, distance to pedestrians, weather conditions, and road type, to inform the decision-making process, determining whether the vehicle must stop, decelerate, or proceed with caution when detecting a potential pedestrian crossing scenario [11]. Conversely, post hoc explanations are applied after AI models, such as DNN or ensemble methods, have been trained. It aims to provide insights into the decision-making processes by analyzing how input features are translated into output decisions in opaque black-box models. Post hoc explanation is critical for applications requiring model transparency, trust, and accountability, specifically when the model’s complexity hinders direct interpretation [216]. Local Interpretable Model-Agnostic Explanations (LIME) is a well-known post hoc explanation technique that approximates the decision-making processes of black-box models by constructing explainable and simplified models within the local vicinity of a specific prediction, thereby allowing stakeholders to gain insight into the reasons behind a model’s decision for a particular input. For example, the authors of reference [218] demonstrated a trust-aware approach for selecting AVs to participate in model training, aiming to ensure system performance and reliability. They utilized the LIME method to calculate the trust values and highlight key features that influenced the selection of each AV during the model training process.

Model dependency, as the name implies, pertains to the extent to which an explanation method is designed for a particular type of ML or DL model or whether it possesses the versatility to be adopted across various model architectures. This concept can be subdivided into (a) model-agnostic techniques and (b) model-specific techniques. Model-agnostic techniques are designed to provide interpretability independent of the underlying architecture of AI models. Model-agnostic methods are extensively utilized owing to their remarkable flexibility and adaptability, which enable them to interpret diverse models and use cases. These methods often provide post hoc explanations and operate by examining the inputs and outputs of an AI model without requiring access to its internal parameters or structures [210,219]. Shapley Additive Explanations (SHAP) serves as a prominent example of a model-agnostic explanation method. SHAP provides valuable insights into the contribution of individual input features to the output of an AI model. Moreover, it facilitates detailed and granular explanations that can either focus on specific individual predictions (local explanations) or provide an overall summary of feature importance across multiple predictions (global explanations) [220]. For instance, the authors of reference [221] proposed WhONet, a wheel odometry neural network that provides continuous positioning information using GNSS data with wheel encoders measurements from the vehicle. The SHAP method was adopted to interpret the predictions of vehicle positioning, thereby enhancing its reliability and ensuring greater transparency and accountability. Contrarily, model-specific techniques are designed to the unique characteristics and architecture of a specific ML or DL model. These methods leverage the intrinsic properties or mathematical properties of the model to provide detailed explanations of its decision-making processes. In other words, model-specific explanation methods require modifications to the explanation framework when applied to different models [216]. Saliency maps exemplify a model-specific interpretability technique that provides pixel-level insights into the significance of input features. This method leverages gradient-based information to identify and highlight the regions of an input (image) that most significantly influence the decision-making processes of an AI model by assigning a salience score to each pixel or region [222]. In other words, a saliency map represents a heatmap that highlights the most visually prominent objects or regions within a given scene. It is imperative to learn that certain studies consider that saliency maps can be generalized to operate in a model-agnostic manner by altering their computation to the model’s input-output behavior rather than its internal gradients [223,224,225]. An illustrative application of saliency maps can be found in reference [226], where the authors proposed a saliency-based object detection algorithm to detect unknown obstacles in autonomous driving environments. This approach integrates the saliency map method into the detection algorithm to amplify image features, thereby emphasizing both known and unknown objects in the environment.

Table 5 and Table 6 below provide a detailed overview of various interpretation techniques that are commonly employed in XAI to improve the interpretability and explainability of AI models. Table 5 categorizes these techniques based on their interpretability level (e.g., local or global), their classification within XAI (e.g., model-agnostic, model-specific, ante hoc, and post hoc), and the types of data they are designed to support. Table 6 presents a comparative analysis, outlining the strengths and limitations of each interpretation technique. By consolidating this information, the tables offer valuable guidance for researchers and practitioners in identifying the most suitable techniques for specific applications. For a more comprehensive exploration of additional interpretation techniques (out of scope in this manuscript), readers are recommended to refer to references [184,188,196,200,201,210,211,216,227,228,229,230,231,232,233].

### 3.2. Roles of XAI in Autonomous Vehicles and Its Challenges

AVs are inherently complex systems, incorporating advanced and intricate AI algorithms to perceive, navigate, and make real-time decisions in dynamic, often unpredictable environments. These decisions necessitate careful consideration of numerous factors, including prevailing traffic conditions, potential road hazards, and interactions with various road users—pedestrians, cyclists, and other vehicles. However, the inherent opacity of sophisticated ML and DL models, often described as the black-box nature of AI, poses significant challenges in translating complex decision-making processes into transparent and understandable explanations, particularly in contexts where trustworthiness, safety, reliability, and accountability are imperative. For example, the rationale behind the decisions to apply brakes or swerve to avoid obstacles during autonomous driving might remain obscure to human stakeholders and may undermine confidence in its reliability and ethical alignment. As a result, the integration of XAI holds paramount importance in addressing these challenges, as it directly impacts critical factors that are essential for the successful deployment, operation, and societal acceptance of these technologies [187].

XAI serves as a critical bridge between advanced AI-driven technologies and human understanding, providing explainable insights into the underlying decision-making processes of AI-driven systems, especially in safety-critical domains such as AVs. One of the primary roles of XAI is to improve transparency, which is a quintessence quality that enables human stakeholders to understand and evaluate the rationale behind the decisions made by autonomous driving systems. It demystifies the black-box nature of intricate AI algorithms and elucidates how inputs, such as sensor data, predetermined rules, and environmental conditions, influence the decisions of acceleration, braking (deceleration), or navigating through complex traffic scenarios. These explanations are often presented using natural language depictions or visualizations, making the decision-making processes of autonomous driving systems more accessible and easier to interpret for diverse audiences [246]. For instance, an XAI-driven multi-sensor perception system of an AV can interpret and elucidate the relative contributions of each sensor in detecting obstacles, such as pedestrians, vehicles, or cyclists, while also providing the underlying rationale for specific decisions such as the decision to decelerate in response to detected hazards. In addition, the system may also integrate visual representations to demonstrate how sensor inputs shaped its decisions, thereby assuring end-users that the vehicle’s decisions and actions are made based on robust and explainable interpretations of its environment. In instances where errors occur, XAI can assist engineers in tracing the decisions back to their originating data sources, which aids in diagnosing issues and improving detection precision and ultimately contributes to the improved transparency and interpretability of the multi-sensor perception system [232,247,248,249].

However, XAI-driven systems still encounter various technical challenges that complicate their implementation and practical usability. Among these, one of the most prominent challenges is the inherent complexity of DL models, which serve as the backbone of many autonomous systems. DL models, especially DNNs, are integral to processing vast amounts of high-dimensional data and making real-time decisions. Nonetheless, their intricate architectures and reliance on sophisticated mathematical computations to achieve optimal performance in driving tasks, such as obstacle avoidance, path planning, and object detection, make it difficult to trace or elucidate the rationale behind a specific output. For example, providing an explanation for why an AV selects a particular route or reacts to hazards in a specific manner in real time often requires advanced interpretability techniques, which are essential to achieving the level of explainability demanded in safety-critical systems for trust and accountability. Thus, achieving an optimal balance between interpretability and model performances remains an ongoing challenge in the development of XAI-driven systems [201]. Other technical challenges involve the need to explain real-time decisions in time-sensitive and safety-critical situations without introducing significant delays that could compromise the system’s performance. For instance, in multi-sensor systems, establishing a unified framework to incorporate multimodal data sources and elucidate the contribution of each sensor modality in real time is a significant challenge as these systems scale in size to address various driving conditions. There is also the potential computational overhead associated with generating interpretable explanations without affecting real-time performances. Moreover, the challenge of establishing a universal explanation technique that applies to diverse and dynamic environments remains significant. This includes the difficulty of explaining decisions made in edge cases or unprecedented conditions, as well as the need to generalize explanations across different driving scenarios, operational contexts, stakeholder groups, and modes of transport (on-road versus off-road) [185,187].

Transparency, in turn, supports trustworthiness, which is a critical factor in promoting the widespread acceptance and successful adoption of AI-driven systems across various domains. In the early stages of technological advancement, machines and algorithms were often viewed as epitomes of trustworthiness and reliability due to their predictability, as their operations and actions were limited to executing predefined tasks that are explicitly programmed, leaving minimal scope for ambiguity or error in their decision-making processes. In recent years, the emergence of ML and DL algorithms has marked a significant paradigm shift, facilitating the creation of systems capable of autonomous reasoning and decision-making. However, this evolution has also introduced an element of unpredictability and opacity into the behavior of AI-driven systems, which in turn undermines the implicit trust due to the underlying complex and opaque reasoning behind their decisions [250]. From end-users’ perspective, the concept of trustworthiness in these systems extends beyond their technical capabilities. It operates as a socio-psychological construct that impacts how individuals, communities, and societies perceive, interact with, and ultimately accept emerging technologies, specifically in autonomous driving systems [251]. One primary factor that affects trustworthiness from a socio-psychological perspective is the fear of the unknown, which stems from the inherent complexity and unpredictability of these technologies. This concern is especially significant in safety-critical applications, where system failure or malfunctions can result in severe and far-reaching consequences. Besides, the lack of clear accountability in autonomous systems intensifies the fear of the unknown, creating significant uncertainty regarding responsibility in the event of system failures or accidents. Thus, the ambiguity surrounding liability and responsibility amplifies public apprehension and undermines trust in AI-driven applications [252]. Other socio-psychological factors influencing the trustworthiness of AI systems include perceived behavioral control, which relates to the user’s capabilities to control or intervene in the system when necessitated, privacy concerns, and perceived usefulness, which refers to the belief that the system will effectively achieve its intended purposes [251]. Thus, it is imperative to highlight transparency and explainability as the foundational elements of trustworthy AI [253].

From a regulatory perspective, the capability to provide explainable insights into AI systems has emerged as an imperative requirement across multiple jurisdictions. As AVs and other AI-driven systems become increasingly integrated into various aspects of society, regulatory authorities have emphasized the critical importance of ensuring transparency and interpretability in their decision-making processes. Thus, the integration of XAI into such applications is important for complying with regulatory mandates and industry standards, as it provides critical mechanisms for comprehending, justifying, and validating the decisions and actions made by AI-driven systems. In addition, it plays an imperative role in supporting transparent investigations and aiding in the determination of liability in the event of an incident [201,254]. In April 2019, the High-Level Expert Group on AI (AI HLEG), appointed by the European Commission (EC), presented a human-centric approach for AI development, which outlines seven ethical guidelines aimed at supporting the development of AI systems that can be considered as trustworthy. Table 7 below outlines the seven ethical guidelines that AI systems must adhere to be deemed trustworthy [250,255,256]. Moreover, XAI is essential in addressing biases within autonomous systems, specifically in instances where such biases stem from unrepresentative training data or flawed algorithmic designs. By enhancing the transparency of the AI decision-making processes, XAI enables the identification and analysis of potential sources of bias that can lead to inequitable or unfair outcomes. This capability ensures that AI-driven systems operate in a fair and unprejudiced manner, thereby preventing the perpetuation of discriminatory practices and promoting unbiased decision-making [179,200]. Nonetheless, one of the ethical challenges of XAI is that it can be challenging to identify the appropriate level of explanation required for different scenarios. Therefore, it is essential to tailor explanations that suit the unique needs and expectations of different uses cases, thereby addressing the distinct requirements of various stakeholders [257]. Furthermore, the ethical challenges associated with data security and data privacy in XAI are significant and multifaceted. It requires an optimal balance between openness and confidentiality, certifying that sensitive data is not compromised or exposed to vulnerabilities while simultaneously ensuring that the explanations provided are clear, interpretable, and meaningful [250,251].

The European Union’s Artificial Intelligence Act, or EU AI Act, which became effective on 1st August, 2024, is implemented to regulate AI systems throughout the European Union (EU) and ensures that AI developed and utilized within the EU is accountable, secure, and transparent with safeguards to protect the fundamental rights of relevant stakeholders [258]. The EU AI Act introduces a risk-based approach to regulating AI and classifies AI systems into four different risk levels: (a) unacceptable risk, (b) high risk, (c) limited risk, and (d) minimal risk. In addition, each risk level is associated with specific requirements that organizations must adhere to in the development or utilization of AI systems. According to the EU AI Act [259], AVs are classified as high-risk AI systems due to their potential impact on public safety and fundamental rights. Consequently, AV manufacturers and in-vehicle software suppliers are obligated to fulfill rigorous requirements to ensure compliance with standards related to transparency, traceability, safety, data governance, and accountability. In reference [260], the author provided a summary of the 12 guiding principles, or commandments, for AV manufacturers and in-vehicle software suppliers in preparing for compliance with the EU AI Act:

1. Assess and classify AI systems adopted in AVs, such as perception, decision-making, or control systems, according to risk.
2. Establish robust risk management systems by developing continuous risk assessment frameworks to evaluate potential AI-related safety risks, including system failures or data biases that could impact the performance of AV systems.
3. Implement robust data governance frameworks to ensure that data used for training AI systems, such as sensor inputs and environmental maps, is of high quality, unbiased, and representative of diverse scenarios.
4. Incorporate mechanisms for human oversight into the design of AV systems, thereby enabling human operators to intervene if the AI system operates in an unpredictable or unsafe manner.
5. Enhance transparency and explainability by systematically documenting system behavior, decision-making processes, and inherent limitations in a manner that is comprehensible to regulators, end-users, and operators.
6. Adopt ethical design and development practices that adhere to the principles of fairness, accountability, and non-discrimination in the creation of in-vehicle software.
7. Develop continuous monitoring and auditing mechanisms to assess and monitor the ongoing post-deployment performance of AVs, which includes regular updates and modifications to ensure continued adherence to safety and performance standards.
8. High-risk AI systems must undergo regular conformity assessments to ensure compliance with all technical, transparency, and safety requirements prior to their introduction to the European market.
9. AI systems should be developed with robust security measures to safeguard against cyber-attacks, system manipulations, or breaches that could compromise the safety of AVs.
10. Incorporate safety mechanisms that prioritize the protection of user well-being, such as collision avoidance, emergency braking, and safeguards for pedestrians and other road users.
11. Maintain detailed records of system development, testing, deployment, and risk assessment, as well as establishing protocols for reporting incidents or system malfunctions to regulatory authorities.
12. The EU AI Act advocates for a human-centric approach to developing AI systems by emphasizing the importance of prioritizing user experiences to increase public trust and acceptability. This involves ensuring that the decision-making processes of AVs are understandable, transparent and intuitive, allowing passengers to feel confident in the AI-based functionalities and autonomous operation of AVs.

Readers interested in the comprehensive overview of the EU AI Act, which comprises 13 chapters, are recommended to refer to reference [259].

## 4. Conclusions and Future Research Recommendations

In this manuscript, we investigated and explored the intersection of multi-sensor fusion and XAI, aiming to address the challenges associated with developing interpretable, trustworthy, and accurate AV systems. We began the survey by introducing the various applications of AVs in both on-road and off-road environments and an overview of the commonly employed sensors integral to developing multi-sensor perception systems, which support critical functionalities, including object detection, obstacle avoidance, and localization and mapping. Subsequently, we presented a comprehensive overview of the various multi-sensor fusion strategies, highlighting their respective strengths and limitations. This offers valuable insights into the various fusion approaches from three primary aspects: (a) when should the sensor fusion occur, (b) where should the sensor fusion occur; and (c) what should the fusion do. Ultimately, selecting the most suitable approaches depends on the specific use cases, requirements, and available resources on the AVs. Additionally, we reviewed some of the cutting-edge multi-sensor fusion techniques and algorithms—traditional and advanced fusion algorithms—discussing their respective applications, strengths, and weaknesses. We also emphasized the challenges involved in the deployment of reliable, safe, scalable, transparent, and comprehensible multi-sensor perception systems in real-world autonomous driving environments. Some of the key challenges are as follows:

- Sensor noise, which relates to the inaccuracies, inconsistencies, or irrelevant data introduced by individual sensors due to a combination of hardware limitations, external interference, or environmental conditions.
- Heterogeneity of sensor modalities in AVs and the resulting system complexity.
- Achieving an optimal balance between accuracy and computational efficiency.
- Multi-sensor fusion systems are susceptible to malicious attacks, which pose significant risk to the integrity and reliability of their autonomous operation.
- Lack of transparency, explainability, and interpretability in black-box AI models, especially in advanced DNN algorithms.

Finally, we explored the core principles of XAI and provided a comprehensive overview of the several emerging XAI strategies and techniques that can be integrated during autonomous systems development to enhance the transparency, trustworthiness, and interpretability of these systems. We summarized the strengths and limitations of these approaches, offering valuable guidance for researchers and practitioners in identifying and selecting the most suitable strategies and methodologies for specific use cases. Moreover, we examined the significance of XAI in AI-driven systems, specifically in AVs, as well as the challenges associated with integrating XAI into real-time autonomous driving applications or other AI-driven technologies. The findings revealed that the lack of interpretability and transparency in advanced AI models, specifically in DNNs, remains a primary challenge due to the opaque, black-box nature of their model architectures and the inherent complexity of these systems. Ultimately, the selection of suitable strategies and methodologies for incorporating XAI depends on the specific system requirements, computational resources, and the associated limitations, all while striving to attain an optimal balance between explainability and system performance. Moreover, several challenges comprising technical, ethical, social, and regulatory aspects remain main issues that must be addressed to enable the successful deployment of XAI systems into real-world environments while ensuring that such systems remain efficient, safe, transparent, trustworthy, and ethical. In addition, we explored the roles of XAI in AV, and the findings showed that the following:

- XAI demystifies the black-box nature of complex AI models adopted in AV systems to improve transparency and interpretability.
- XAI supports trustworthiness, which is a critical factor in increasing public trust and the widespread acceptance and successful adoption of AI-driven systems.
- XAI supports transparent investigations and aids in the determination of accountability in the event of an accident.
- XAI provides a critical mechanism to comprehend, justify, and validate the decisions and actions made by AI-driven systems, thereby facilitating compliance with regulatory requirements and industry standards.
- XAI supports safety assurance to end-users by explaining decisions in critical scenarios during autonomous operations.
- XAI ensures that AV adheres to ethical principles and assists stakeholders in evaluating and understanding the ethical trade-offs in scenarios like collision avoidance.
- XAI facilitates the iterative model improvement and refinement by providing transparent insights into the underlying behavior of AV systems.

In summary, the development of methodologies that ensure real-time explainability for stakeholders without compromising safety and accuracy is paramount in the successful deployment of AVs and other AI-driven systems. It ensures that stakeholders, including end-users, engineers, operators, and regulators, can understand the reasoning behind critical decisions during operation in complex and dynamic environments, fostering trust and enabling timely interventions when needed. Nonetheless, it is essential to customize the explanations to meet the specific needs and expectations of different use cases, thereby addressing the diverse requirements of various stakeholders. In autonomous driving, vehicles operate in real time and must adapt to rapidly changing situations. It is imperative to attain an optimal balance between the computational requirements necessitated for accurate real-time decision-making and the need for explainability and transparency without introducing delays that could result in potential hazardous outcomes. Hence, it is essential to develop efficient and scalable XAI methods that provide clear, comprehensible, and real-time explanations while maintaining the operational safety and decision-making accuracy of autonomous systems. Such methods are critical for fostering trust and accountability, aiding in error diagnosis, ensuring compliance with regulatory requirements, and supporting the ethical and responsible integration and deployment of autonomous technologies into real-world environments.

Future research directions aimed at progressing the integration of XAI into real-time, high-stakes AVs or other AI-driven systems encompass a range of innovative and critical domains. Such explorations aim to address existing challenges and unlock new opportunities to enhance the safety, reliability, interpretability, transparency, and trustworthiness of these systems. A significant area of focus for future research involves the development of a unified context-aware evaluation framework for comparing and selecting interpretability techniques across multiple domains or, at a minimum, achieving uniformity within specialized areas. This could contribute to the development of best practices in XAI, providing valuable, contextual, and adaptive insights that are aligned with specific goals, stakeholders, and operational constraints of different domains—cross-disciplinary, human–AI collaboration [187]. Over time, this would support the development of more transparent, reliable, and user-centric AI systems [201,261,262]. Moreover, it is essential to investigate and develop novel XAI approaches that facilitate the provision of accurate and computationally efficient real-time explanations, specifically in memory-constrained, real-time industrial systems like autonomous driving and healthcare [188]. Another promising direction for future research involves integrating causal relationships into XAI, with the objective of enhancing the capability of AI systems to offer more comprehensive explanations for their decisions. This approach aims to elucidate the underlying causal factors that impact the outcomes, thereby enabling a transparent understanding of the cause-and-effect dynamics involved in the decision-making process [188,233,263,264].

Furthermore, it is important to investigate and refine cutting-edge multi-sensor fusion algorithms capable of processing and interpreting large-scale sensor data in real time. Such advancements are vital to ensuring the accuracy and reliability of autonomous systems in dynamic driving environments while simultaneously providing clear and understandable explanations of the underlying decision-making process. From an ethical and regulatory perspective, future research should prioritize the development of methodologies aimed at incorporating fairness, non-discrimination, and privacy protections into AI systems. Simultaneously, it is vital to ensure that these systems comply with emerging ethical and regulatory standards, thereby fostering trust and accountability within AI technology [188,265]. Other future research avenues may involve incorporating large language models (LLMs) to aid in the generation of clear, contextually relevant, and user-friendly explanations for various stakeholders, including passengers, regulators, and legal professionals [266,267]. Besides, investigating the different methodologies for preventing adversarial attacks is vital in ensuring the security and integrity of AI systems, specifically in safety-critical applications [256,268,269]. Finally, improving the knowledge and skills of practitioners and researchers in XAI through continuous education and training will significantly contribute to the advancement of interpretability research and its practical applications. It is also important to develop accessible and effective educational frameworks aimed at fostering public understanding of AI systems, their capabilities and limitations, and their decision-making processes [201,251]. We hope that these research avenues will facilitate the development of AI models that are reliable, trustworthy, interpretable, and safe, thereby advancing the field of XAI and enhancing transparency and interpretability in AVs.

## Figures and Tables

**Table 1:** A summary of the commonly utilized proprioceptive and exteroceptive sensors in AVs.

	Definition	Examples
Exteroceptive Sensor	It perceives the external environment, detecting objects, obstacles, light intensity, and other relevant features essential for safe navigation.	Vision cameras. Radar sensors. Lidar sensors. Ultrasonic sensors.
Proprioceptive Sensor	It measures the internal values and gathers information about the dynamic state of a self-driving vehicles, such as its position, speed, and acceleration, that are essential for maintaining stability and ensuring precise control of the vehicle motion.	IMU. Global Navigation Satellite System (GNSS). GPS.

**Table 2:** An overview of the advantages and limitations associated with exteroceptive sensors: camera, Lidar, radar, and ultrasonic sensors. The table shown is adapted from [49] with modifications.

Exteroceptive Sensors	Advantages	Disadvantages
Lidar	Long detection range. Provides high-resolution 3D spatial data with distance measurements. Insusceptible to illumination. Performs well in diverse environmental conditions, but it depends on the wavelength and system design [50].	Difficult to detect objects with specular surface or non-Lambertian material [51]. No direct measurement of velocities by conventional time-of-flight (ToF) Lidars [52]. Susceptible to interference or cross-talk [53,54]. Limited performance in strong ambient light conditions [55,56]. Ineffective and shorter range in adverse heavy rain, fog, or dust. No texture or color information. High cost.
Camera	High resolution. Infrared or thermal sensing available. Captures texture and color details. Optimal for object recognition. Low cost.	Depth information is not possible without stereo configuration. Reliant on illumination. Vulnerable to weather conditions. Extensive computational power required to analyze camera images. Limited velocity measurements.
Radar	Insusceptible to illumination and weather conditions. Offers distance and relative velocity measurements. Low cost. Long range.	Poor resolutions. Unable to detect small objects. Limited classification capability. Noisy outputs due to reflections. No texture or color information.
Ultrasonic	Insusceptible to illumination and weather conditions. Provides high precision for close-range detection at low speed. Capable of detecting objects made from all types of materials. Low cost.	Limited detection range. Not suitable for detecting objects at high speed. Susceptible to interference from wind at high speed. Sensitive to temperature variation and vapors.

**Table 3:** An overview of the pros and cons associated with centralized fusion, decentralized fusion, and distributed fusion [49,81,82,83].

	Advantages	Disadvantages
Centralized Fusion	Easy to maintain and update as all data processing occurs in the central processing unit. High processing power. Can leverage advanced processing techniques and complex algorithms that require significant computational resources without the need for synchronization across multiple nodes. Efficient multi-sensor data fusion as all data is integrated at a single central processor.	High computational load on the central processor, potentially leading to latency issues. Single point of failure. Limited scalability as it can create bottlenecks in both data transmissions and processing power as the number of sensors increases. Limited bandwidth, especially in high-speed or resource-constrained systems.
Decentralized Fusion	Reduces computational burden on a single processor by distributing processing tasks across multiple nodes. Robust to failure of individual processing units or one node. Improves scalability where the system can handle additional sensor modalities without overloading the central processor. Reduces communication delays and enable faster decision-making by enabling parallel data processing.	Complex communication and synchronization can lead to delays or conflicts during data fusion. Risk of data inconsistency if synchronization is handled ineffectively. Data redundancy as multiple sensors may perform similar processing tasks independently. Limited computational resources for individual nodes to process large amounts of data compared to a central processing unit.
Distributed Fusion	Improves robustness and fault tolerance as the failure of one node or sensor does not compromise the entire system. Enables faster decision-making as local processing can occur in parallel across different nodes. Reduces potential bottlenecks and latency. Flexible and adaptive to changing environments or multi-sensor configurations.	Requires effective coordination and communication protocols between distributed nodes to ensure seamless integration and synchronization of data. Increased complexity in data management and fusion due to the distributed nature of the system. Computational and communication overhead in real-time, large scale, resource-limited systems.

**Table 4:** An overview of the advantages and limitations of traditional and advanced learning algorithms employed in multi-sensor fusion systems for AV applications, such as the Unscented Kalman Filter (UKF) algorithm, Particle Filter (PF) algorithm, Dempster–Shafer Theory (DST), YOLO convolutional neural network (CNN), PointNet, and Faster R-CNN.

Algorithms	Descriptions	Applications	Ref.
UKF	UKF is an advanced adaptation of the KF algorithm, specifically developed to address nonlinearities in state estimation with greater efficiency and accuracy. Its strengths and limitations include the following: Improved accuracy in nonlinear systems. Less susceptible to divergence in scenarios where linear approximations might fail. High computational overhead in high-dimensional systems. Sensitive to noise modelling. Requires careful initialization of parameters for optimal performance. Requires prior knowledge of systems model and data.	Simultaneous Localization and Mapping (SLAM). Object tracking.	[137,138,141]
Particle Filter (PF)	PF is a recursive algorithm that is utilized to estimate the state of a system by using a set of random samples (particles) to represent the probability distribution, making it ideal for nonlinear and non-Gaussian problems. Its strengths and limitations include the following: Highly effective for systems with nonlinear dynamics and non-Gaussian noise. Scalable for real-time applications with optimization. Flexible and can integrate data from multiple sensor modalities. Prone to particle degeneracy. Sensitive to initial particle distribution, and improper initialization can lead to inaccurate estimates. High computational cost.	Object tracking. Trajectory prediction. Localization.	[142,143,145]
Dempster–Shafer Theory (DST)	DST is a mathematical framework for modeling uncertainties in real-world problems and combining evidence from different sources to make decisions, even if that evidence is uncertain or incomplete, to form a belief about a hypothesis. Its strengths and limitations include the following: Does not require pre-defined probabilities. Integrates evidence from diverse sources with varying reliability. Improves decision-making by representing varying levels of belief. Computational expensive in large systems. Struggles with conflicting evidence. May produce high uncertainty in complex, high-dimensional data.	Object fusion detection. Tracking dynamic objects. Classification. Decision-making in complex environments.	[139,146,147]
YOLO	YOLO is a real-time object detection algorithm that utilizes a single CNN (single-stage detector) to predict bounding boxes and class probabilities from an image. Several versions of YOLO have been established, each offering improved precision, with the most recent version being YOLOv11 [156]. Its strengths and limitations include the following: Fast and able to handle multi-scale object detection in real time. Offers high precision in object localization and classification. Does not require manual feature extraction. Less accurate than other methods due to coarse bounding boxes. High computational cost, especially in high-resolution images. Poor detection of occluded objects and small targets.	Real-time object detection. Traffic sign recognition.	[11,134,140,148]
Faster R-CNN	Faster Region-Convolutional Neural Network (Faster R-CNN) is a two-stage object detection algorithm that utilizes a Region Proposal Network (RPN) and a CNN to detect and localize objects in complex real-world images. Its strengths and limitations include the following: High detection precision. Performs well in cluttered or occluded environments. Combines region proposal and object classification in a unified framework (end-to-end training). Requires significant computational resources for training and inference. Degraded performance when detecting small objects in dense environments. Slow inference time, which can be challenging for real-time applications.	Real time object detection.	[102,140,149,150,151]
PointNet	PointNet is a two-stage detector that introduces a permutation-variant deep neural network to learn global features from unordered point clouds using a symmetric function, without the need for voxelization. Its strengths and limitations include the following: Handles unordered point cloud data. Can learn directly from raw data without feature engineering. Sensitive to noisy or sparse data. Limitations in generalizing to new or unseen scene configurations. Lack of fine-grained feature extraction but PointNet++ [157] is introduced to address this limitation.	3D object detection. Semantic segmentation. Localization. Obstacle detection and avoidance.	[152,153,154,155]

**Table 5:** An overview of interpretation techniques for XAI. These techniques are categorized based on their interpretability level (e.g., local or global), their explainability classification (e.g., ante hoc, post hoc, model-agnostic, and model-specific), and the types of input data (e.g., unstructured data—textual data, structured data—tabular, and image) that each technique can handle. The acronyms from top to bottom at the first column are as follows: BRL—Bayesian Rule Lists; GAM—Generalized Additive Model; LIME—Local Interpretable Model-Agnostic Explanations; SHAP—Shapley Additive Explanation; Grad-CAM—Gradient-weighted Class Activation Mapping; DeepLIFT—Deep Learning Important Features; PDP—Partial Dependence Plot. This table has been adapted and revised based on references [184,188,200,201,210,211,216,227,228,229,230,231,233].

Techniques	Explanation-Level		Implementation-Level		Model Dependency		Data Type		
	Global	Local	Ante hoc	Post hoc	Agnostic	Specific	Tabular	Image	Textual
Decision Tree	●	●	●	-	●	-	●	-	-
Linear Model	●	-	●	-	●	-	●	-	-
BRL	●	-	●	-	-	●	●	-	-
GAM	●	-	●	-	-	●	●	-	-
LIME	-	●	-	●	●	-	●	●	●
SHAP	●	●	-	●	●	-	●	●	●
Saliency Maps *	-	●	-	●	●	●	-	●	-
Grad-CAM	-	●	-	●	●	-	-	●	-
Anchors	-	●	-	●	●	-	●	●	●
DeepLIFT	●	●	-	●	●	-	-	●	●
Counterfactuals	-	●	-	●	●	-	●	●	●
Sensitivity Analysis *	●	-	-	●	●	●	●	-	-
Distillation	●	-	-	●	-	●	●	●	●
PDP	●	●	-	-	●	-	●	-	-
Feature Importance	●	●	-	●	●	-	●	●	●

* Saliency maps and sensitivity analysis can be adapted to function in a model-agnostic manner by modifying their computation to focus on the input-output relationships of a model [223,224,225,231].

**Table 6:** A comparative analysis of interpretation techniques, highlighting their respective strengths and limitations. This table has been revised and adapted based on references [184,188,200,201,210,216,217,218,227,228,229,230,231,232,233]. The acronyms from top to bottom (first column) are BRL—Bayesian Rule Lists; GAM—Generalized Additive Model; LIME—Local Interpretable Model-Agnostic Explanations; SHAP—Shapley Additive Explanations; Grad-CAM—Gradient-weighted Class Activation Mappings; DeepLIFT—Deep Learning Important Features; PDP—Partial Dependence Plot.

Techniques	Strengths	Limitations
Decision Tree	Easy to understand. Robust to outliers and missing values. High interpretability. Able to handle non-linear relationships.	Lack of stability, where small changes in training data can result in significant variations. Prone to overfitting. Non-smooth decision boundaries. Not applicable to linear relationships.
Linear Model	Simple and easy to implement. Computationally inexpensive. Generalize well to new datasets with linear relationships. Transparent, no hidden layers or complex transformations.	Not applicable to non-linear relationships. Oversimplified explanations may not be sufficient for safety-critical applications. Coefficients of linear models become unstable and unreliable when input features are highly correlated. Sensitive to outliers.
BRL	The IF-THEN rules are easy to interpret. Incorporation of prior knowledge, which can guide the learning process and improve model performance. Automatic feature selection. Can handle noisy and incomplete data by modeling uncertainty.	High computational cost. Difficult to model complex and high-dimensional environments. Sensitive to noisy or incomplete data. Not feasible for large-scale systems due to scalability issues.
GAM	Flexible—can handle linear and non-linear relationships in data. No black-box nature. Provides clear and interpretable relationships between input features and predicted output. Can include regularization techniques to control model complexity.	Computationally intensive in large datasets or high-dimensional data. Sensitive to smoothing parameters. Require large sample sizes to capture non-linear patterns effectively [212]. Risk of overfitting in highly complex data.
LIME	Computationally efficient. Simple and intuitive for local interpretation. Flexible; can be applied to any ML models. Works well on tabular, image, and text data.	Lacks precision in capturing global feature importance. Sensitive to perturbations and may require hyperparameter tuning [234]. Sensitive to small changes in data or the neighborhood around the instance [235]. Limited to local context.
SHAP	Versatile—can be applied to various ML models [236]. Provides more accurate explanations than LIME. Fair attribution to prevent biased explanations. Can handle simple and complex models.	High computational cost. Can be manipulated by adversarial attacks [237]. May require approximations in large, complex DNNs that can reduce accuracy. Assume feature independence.
Saliency Maps	Intuitive visualization. Can be applied during model inference. Effective in explaining decisions of image-based models, such as CNN. Supports model debugging.	Limited to gradient-based models Sensitive to noise. Lack of global interpretability. Requires backpropagation, which can be computationally expensive. Can be manipulated by adversarial attacks [238].
Grad-CAM	Intuitive visual explanations. Localized insights. Robust to adversarial perturbations in image classification tasks. Supports model debugging by highlighting which areas of the input are important for predictions.	Lacks ability to highlight fine-grained details. Computationally expensive to calculate gradients in deep models. Does not effectively localize objects within an image when multiple instances of the same class are present [239].
Anchors	Less computation than SHAP. Better generalizability than LIME [240]. Can be applied to any ML models regardless of its architecture. High fidelity.	Require tuning to provide optimal explanations [241]. Requires discretization and highly configurable and impactful setup. Computationally intensive.
DeepLIFT	Compatible with DNN. Efficient explanation generation. Captures complex interactions between features. Scalable. Local and global interpretability.	Sensitive to initialization. Depends on a reference point or baseline, which might not always be appropriate in certain contexts. Produces inconsistent results due to redefining gradients. Struggles to offer global explanations for more complex and ensemble models.
Counterfactuals	User centric—provides intuitive explanations with “what-if” scenarios. Does not require access to the data or the model. Easy to implement. Provides actionable insights.	High computational cost in high-dimensional models. Ambiguity in interpretation and may require expert judgement in specific contexts. Potential risk of neglecting complex relationships in data. Inability to capture all aspects of model behavior, limiting the comprehensiveness of the explanation.
Sensitivity Analysis	Provides intuitive explanations. Provides unique solution, training-free process, and fast computation [242]. Identifies weak and prominent features. Applicable to various model types without requiring access to internal parameters.	Limited to global insights. Require explicit modeling of complex feature interactions Computationally expensive for complex models due to multiple evaluations for each input variation. Generates noisy explanation maps
Distillation	Simplifies complex models. Can be applied across various ML models. Does not require the creation of additional rules or decision pathways. Maintains model’s performance while ensuring interpretability.	Dependence on the teacher model (complex model). Potential loss of fine-grained details during compression. Sensitive to hyperparameters. Increased computational cost.
PDP	Easy to implement. Provides clear and causal interpretation. Offers intuitive visualization. Delivers global insights into the overall impact of individual features on predictions.	Assumes no correlation between features. High computational cost in large datasets. Restricted to marginal effects, showing the influence of a maximum of three features at once. Potential to overlook heterogeneous effects.
Feature Importance	Provides clear and intuitive explanations. Identifies critical factors influencing decision-making [243]. Aids in model debugging by detecting potential biases, errors, or overfitting through feature analysis. Offers flexibility as a model agnostic approach.	Complex feature interactions may be overlooked when decision-making processes are overly simplified. Feature importance values are context-dependent and may vary significantly across different data distributions or conditions. High computational cost for large models. Over-reliance on the assumption of feature independence, not suitable in scenarios where features are correlated [244,245].

**Table 7:** An overview of the seven essential criteria outlined in the established ethical guidelines by the High-Level Expert Group on AI (AI HLEG) that AI systems must follow to be deemed as trustworthy. This table has been revised and adapted based on references [250,255,256].

Criteria	Explanations
Human Agency and Oversight	AI systems should enhance human decision-making and support fundamental rights while ensuring adequate oversight, rather than restricting or misleading human autonomy. This can be achieved through human-in-the-loop, human-on-the-loop, and human-in-command approaches.
Technical Robustness and Safety	AI systems must be resilient, secure, and safe, with contingency plans in place to address system failures or malfunctions. They must also be accurate, reliable, and reproducible to minimize and prevent unintentional harm.
Privacy and Data Governance	In addition to safeguarding privacy and data protection, effective data governance mechanisms must be established, ensuring data quality, integrity, and authorized access. End-users should also maintain full control over their personal information, ensuring that such data is not used in ways that could be detrimental or harmful to their interests.
Transparency	Data, systems, and AI business models must be transparent, with traceability mechanisms ensuring accountability. Moreover, AI systems and their decisions should be explained in a way that is tailored to the relevant stakeholders, and it is essential that users are aware that they are interacting with AI and are informed of its capabilities and limitations.
Diversity, Non-Discrimination, and Fairness	Unfair bias must be eliminated to prevent negative outcomes such as the marginalization of vulnerable groups and the reinforcement of prejudice. AI systems should be accessible to all, regardless of disability, and involve relevant stakeholders throughout their lifecycle to promote inclusivity.
Societal and Environmental Well-Being	AI systems must be designed to benefit all humanity, including future generations, while prioritizing sustainability and environmental responsibility. Additionally, their impact on the environment, other living beings, and society must be thoroughly evaluated and considered.
Accountability	Mechanisms must be established to ensure accountability for AI systems and their outcomes. Auditability, which allows for the evaluation of algorithms, data, and design processes, is essential, particularly in critical applications. Additionally, accessible avenues for compensation should be provided.

## Data Availability

No new data were created or analyzed in this study. Data sharing is not applicable to this article.

## Abbreviations

The following abbreviations are used in this manuscript:

3D	Three dimensional
AI	Artificial intelligence
AI HLEG	High-Level Expert Group on AI
AV	Autonomous vehicles
BEV	Bird’s-eye view
BRL	Bayesian rule lists
CNN	Convolutional Neural Networks
DeepLIFT	Deep Learning Important Features
DL	Deep learning
DNN	Deep Neural Network
DST	Dempster–Shafer Theory
EC	European Commission
EM	Expectation-maximization
EU	European Union
Faster R-CNN	Faster Region-Convolutional Neural Network
GAM	Generalized Additive Model
GNSS	Global Navigation Satellite System
GPS	Global Positioning System
GPU	Graphics processing unit
Grad-CAM	Gradient-weighted Class Activation Mapping
HD	High-definition
HLF	High-level fusion
IMU	Inertial Measurement Unit
IoU	Intersection over Union
KF	Kalman Filter
KNN	K-Nearest Neighbors
LIME	Local Interpretable Model-Agnostic Explanations
LLF	Low-level fusion
LLM	Large language model
ML	Machine learning
MLF	Mid-level fusion
MMLF	Mult-modal multi-class late fusion
NMS	Non-Maximum Suppression
PDP	Partial Dependency Plots
PF	Particle Filter
RBM	Restricted Boltzmann Machine
RL	Reinforcement learning
RMG	Rail-Mounted Gantry
RNN	Recurrent Neural Networks
RPN	Region Proposal Network
SAE	Society of Automation Engineers
SCFT	Spatio-Contextual Fusion Transformer
SHAP	Shapley Additive Explanations
SPA	Soft Polar Association
ToF	Time-of-Flight
TPU	Tensor Processing Unit
UKF	Unscented Kalman Filter
XAI	Explainable artificial intelligence
